# A Computational Assessment of Target Engagement in the Treatment of Auditory Hallucinations with Transcranial Direct Current Stimulation

**DOI:** 10.3389/fpsyt.2018.00048

**Published:** 2018-02-22

**Authors:** Won Hee Lee, Nigel I. Kennedy, Marom Bikson, Sophia Frangou

**Affiliations:** ^1^Department of Psychiatry, Icahn School of Medicine at Mount Sinai, New York, NY, United States; ^2^Department of Biomedical Engineering, The City College of New York, City University of New York, New York, NY, United States

**Keywords:** auditory hallucinations, neuroimaging, computational modeling, transcranial direct current stimulation, schizophrenia

## Abstract

We use auditory verbal hallucinations (AVH) to illustrate the challenges in defining and assessing target engagement in the context of transcranial direct current stimulation (tDCS) for psychiatric disorders. We defined the target network as the cluster of regions of interest (ROIs) that are consistently implicated in AVH based on the conjunction of multimodal meta-analytic neuroimaging data. These were prescribed in the New York Head (a population derived model) and head models of four single individuals. We appraised two potential measures of target engagement, tDCS-induced peak electric field strength and tDCS-modulated volume defined as the percentage of the volume of the AVH network exposed to electric field magnitude stronger than the postulated threshold for neuronal excitability. We examined a left unilateral (LUL) montage targeting the prefrontal cortex (PFC) and temporoparietal junction (TPJ), a bilateral (BL) prefrontal montage, and a 2 × 1 montage targeting the left PFC and the TPJ bilaterally. Using computational modeling, we estimated the peak electric field strength and modulated volume induced by each montage for current amplitudes ranging 1–4 mA. We found that the LUL montage was inferior to both other montages in terms of peak electric field strength in right-sided AVH-ROIs. The BL montage was inferior to both other montages in terms of modulated volume of the left-sided AVH-ROIs. As the modulated volume is non-linear, its variability between montages reduced for current amplitudes above 3 mA. These findings illustrate how computational target engagement for tDCS can be tailored to specific networks and provide a principled approach for future study design.

## Introduction

Transcranial direct current stimulation (tDCS) is a non-invasive neuromodulation technique currently under evaluation for the treatment of neuropsychiatric conditions. tDCS involves the application of a weak electric current that flows through the brain from anodal to cathodal scalp electrodes. Proposed mechanisms of action for tDCS involve polarity-dependent changes in the resting membrane potential (depolarization at the anode and hyperpolarization at the cathode) and changes in synaptic plasticity (1–5).

As the application of tDCS to neuropsychiatric disorders is expanding, the field has begun to recognize significant conceptual gaps that hamper the design and evaluation of tDCS treatment protocols. In particular, there are currently no assays for assessing target engagement in tDCS studies, which makes it very difficult to discern the basis for therapeutic efficacy or lack thereof. This contrasts with drug development, where measures of target engagement are an essential and integral part of study design. In human studies, the *in vivo* assessment of drugs is critically dependent on proximal markers (e.g., receptor occupancy) as these enable a direct correlation between target engagement and measurements of drug efficacy or toxicity (6). In tDCS, target engagement depends on patient-specific factors related to head anatomy and functional state and on operator-controlled factors related to stimulation parameters (montage, current amplitude) and administration protocol (frequency and duration of treatment sessions) (7). In psychiatry, an additional challenge involves uncertainties in the definition of the target brain regions as a direct and consistent correspondence between symptoms and brain networks has yet to be established (8).

Here, we present an approach for the assessment of target engagement measures in tDCS studies of psychiatric symptoms. We focus on auditory verbal hallucinations (AVH) in patients with schizophrenia because the neural correlates of AVH are arguably better defined than those of other psychiatric symptoms. Current models of AVH propose a dual pathology involving reduced cognitive control due to hypofunction of prefrontal cortex (PFC) and abnormal activation of speech-related regions, in the superior temporal gyrus and temporoparietal junction (TPJ) (9, 10). This model has informed the tDCS montages that have been used to treat AVH that conventionally target these two regions. Despite initial positive results in case series and open label trials (Table S1 in Supplementary Material), randomized, double-blind, sham-controlled clinical trials (RCTs) have not been consistently differentiated between the active tDCS and the sham condition (11–15). Without methods to assess tDCS target engagement, it is difficult to interpret these results and improve study design.

In response, we outline an approach for improving the definition of the target network for AVH and for the assessment of two measures that could putatively provide a quantitative assessment of target engagement in tDCS. First, we identified the brain regions that comprise the AVH network using data from meta-analyses of the relevant structural and functional imaging studies. Second, to accommodate concerns about anatomical variation, we prescribed the brain regions in the AVH network as regions of interest (ROIs) in a population-based standardized volume conductor head model and in four-head models from single individuals. We then used computational modeling to quantify two putative measures of target engagement, peak electric field strength, and percentage-modulated volume derived from three different tDCS protocols. Peak electric field strength in AVH-network regions was chosen as it tracks cortical excitability in tDCS studies of motor regions (16, 17). We introduced modulated volume as another potential index of target engagement. As AVH involve a network of brain regions (as opposed to a single region like the motor cortex), the efficacy of tDCS may depend on the percentage of the volume of the network being modulated. Our primary aim was to quantify the degree of engagement of the AVH network by different tDCS montages based on the peak electric field strength and percentage-modulated brain volume. Our secondary aim was to evaluate the degree of variation in these as a function of the anatomical variability of the head models used. For modulated volume, which is a non-linear measure, we also examined interindividual variability for each montage as a function of increasing current amplitude within the tolerable current range of 1–4 mA (18). Taken together, these steps outline a novel approach to the design of tDCS interventions for target identification and engagement that is image-guided, multi-target, and supports rational testing of therapeutic hypotheses.

## Materials and Methods

### Identification of the AVH Network in the Brain

We interrogated databases available through the National Center for Biotechnology using relevant expanded subject headings and free text searches to identify meta-analyses of structural and functional neuroimaging studies of AVH published by February 1, 2017. Coordinates of AVH correlates reported in Talairach space were transformed to Montreal Neurological Institute space, using the “tal2icbm_fsl” transform.^1^ All coordinates were mapped to the Automated Anatomical Labeling digital atlas (19) to identify anatomical regions of cross-modal convergence. The regions thus identified comprise the target regions of interest (AVH-ROIs) used in subsequent computational models.

### Prescription of AVH-ROIs in Population-Level and Individual Head Models

Based on the meta-analytic evidence described earlier, we prescribed the AVH-ROIs identified in five previously developed three-dimensional (3D) realistic models of the human head (20–22). These comprised the New York Head (S1) and four head models belonging to single individuals (S2–S5). The New York Head (S1) is a publicly available standardized volume conductor head model (0.5 mm^3^ isotropic resolution) that was constructed using T1-weighted MRI scans of 152 adult human brains acquired at 0.5 mm^3^ isotropic resolution and segmented into 6 tissue compartments comprising the skin, skull, cerebrospinal fluid (CSF), gray matter, white matter, and air cavities (20). The individual head models belonged to three men (S2, S3, and S4; aged 34–41 years) (21–23) and one woman (S5; aged 34 years) (22). They were generated from structural T1-weighted MRI scan with 1 mm^3^ isotropic resolution and segmented into the same six tissue compartments as the New York Head. These models are available upon request. For S2, individual tissue probability maps corresponding to gray matter, white matter, and CSF were automatically created using FAST (FMRIB Analysis Group, Oxford, UK) (24). The non-brain regions were semiautomatically segmented into three tissue compartments including skin, skull, and air using an in-house segmentation algorithm (21, 25–27), followed by correction of segmentation errors using tools from ITK-SNAP (28). For S3–S5, automated segmentation was performed using SPM (29), followed by correction of segmentation errors using an in-house MATLAB script (30) and ScanIP tools (Simpleware Ltd., Exeter, UK).

### tDCS Electrode Montages

The search space for optimal electrode placement in the treatment of AVH is too vast for systematic empirical evaluation. Taking a pragmatic approach, we focused on three montages: (A) a left unilateral (LUL) montage with anode over the left PFC (F3-FP1) and cathode over the left TPJ (P3), (B) a bilateral (BL) prefrontal 1 × 1 montage with the anode over the left PFC (F3-FP1) and cathode over the right PFC (FP2), and (C) a third montage (2 × 1) with one anode over the left dorsolateral PFC (F3-FP1) and two cathodes, one on the left and the other on the right TPJ (T3-P3 and T4-P4). The first two montages have been most widely used in tDCS studies (Tables S1 and S2 in Supplementary Material), while the third was included because of its theoretical potential to provide better target engagement of right-sided target AVH-ROIs.

### Electric Field Computational Modeling

Computational modeling was performed separately for each of the three montages in each of the five head models. In addition, we assessed the effect of varying current amplitude across all three montages and head models within the range of 1–4 mA, which is known to be tolerable in humans (31). We used the same computational modeling approach based on our previous work (21, 26). The tDCS sponge electrodes were modeled with 5 cm × 7 cm surface intersecting the scalp. For each montage, the complete 3D head models incorporating the tDCS electrodes were adaptively tessellated to produce finite element models using the restricted Delaunay triangulation algorithm (32). The electric conductivities used for each tissue type across all five head models were as follows: gray matter = 0.33 S/m, white matter = 0.14 S/m, CSF = 1.79 S/m, skin = 0.43 S/m, skull = 0.0132 S/m, and air = 0 S/m (21, 23, 27, 33, 34). The electrodes were assumed to have the conductivity of saline (1.4 S/m) (22). Constant electric current was applied to the electrode surfaces away from the head (26). The LUL and BL montages correspond to those that have been used in the current literature, and accordingly current intensity was set at 2 mA, unless otherwise stated (Table S1 in Supplementary Material). For the 2 × 1 montage, 2 mA was applied to the frontal anode and −1 mA to each of the posterior cathodes, unless otherwise stated. The electric field distribution was computed by solving the Laplace equation using the preconditioned conjugate solver within ANSYS (ANSYS Inc., Canonsburg, PA, USA) (21, 26). In addition, we modeled radial cortical electric field that represents the inward and outward component of the electric field relative to the cortical surface (22).

### Data Analysis

We computed the electric field magnitude (*E*) and the percentage modulated volume for each AVH-ROI. The modulated brain volume was defined as the percentage of the brain volume (within the left or right AVH networks) exposed to electric field magnitude (*E*) stronger than the modulation threshold (*E*_th_), i.e., the volume where *E*/*E*_th_ ≥ 1 (21, 34). We acknowledge that the modulation threshold in living humans is yet to be conclusively determined. For these analyses, we used a published estimate of the modulation threshold (*E*_th_ = 0.2 V/m) based on previous evidence regarding the minimum electric field strength likely to change the firing rate of neurons in model *in vitro* systems (35). Several studies have tried to define the minimum applied electric field sufficient to modulate transmembrane potential using different methodologies involving *in vitro* recordings from single neurons and brain slices and *in vivo* recordings in animals (36–38); the values reported in the case of direct current stimulation ranged from 0.18 to 0.5 V/m, with 0.2 V/m being most commonly reported. We therefore chose this threshold which theoretically should yield the maximum modulated brain volume for each montage. Subsequently, interindividual variation in peak electric field strength and modulated brain volume across the five head models were determined using coefficient of variation (CV). To examine the effect on the modulated brain volume of altering the current amplitude, the electric field simulations for all montages were scaled linearly to span the range of 1–4 mA and the volume where *E*/*E*_th_ ≥ 1 was estimated for these ranges. Finally, we used analyses of variance, followed by Bonferroni-corrected *post hoc* pairwise comparisons, to determine if the peak electric field strength and modulated brain volume in the AVH-ROIs differed across the three tDCS montages. The effect size for the peak electric field strength and modulated brain volume between each pair of electrode montages was computed using the Cohen’s *d* (39).

## Results

### Identification and Prescription of the Target Brain Network for AVH

**Figure 1:**
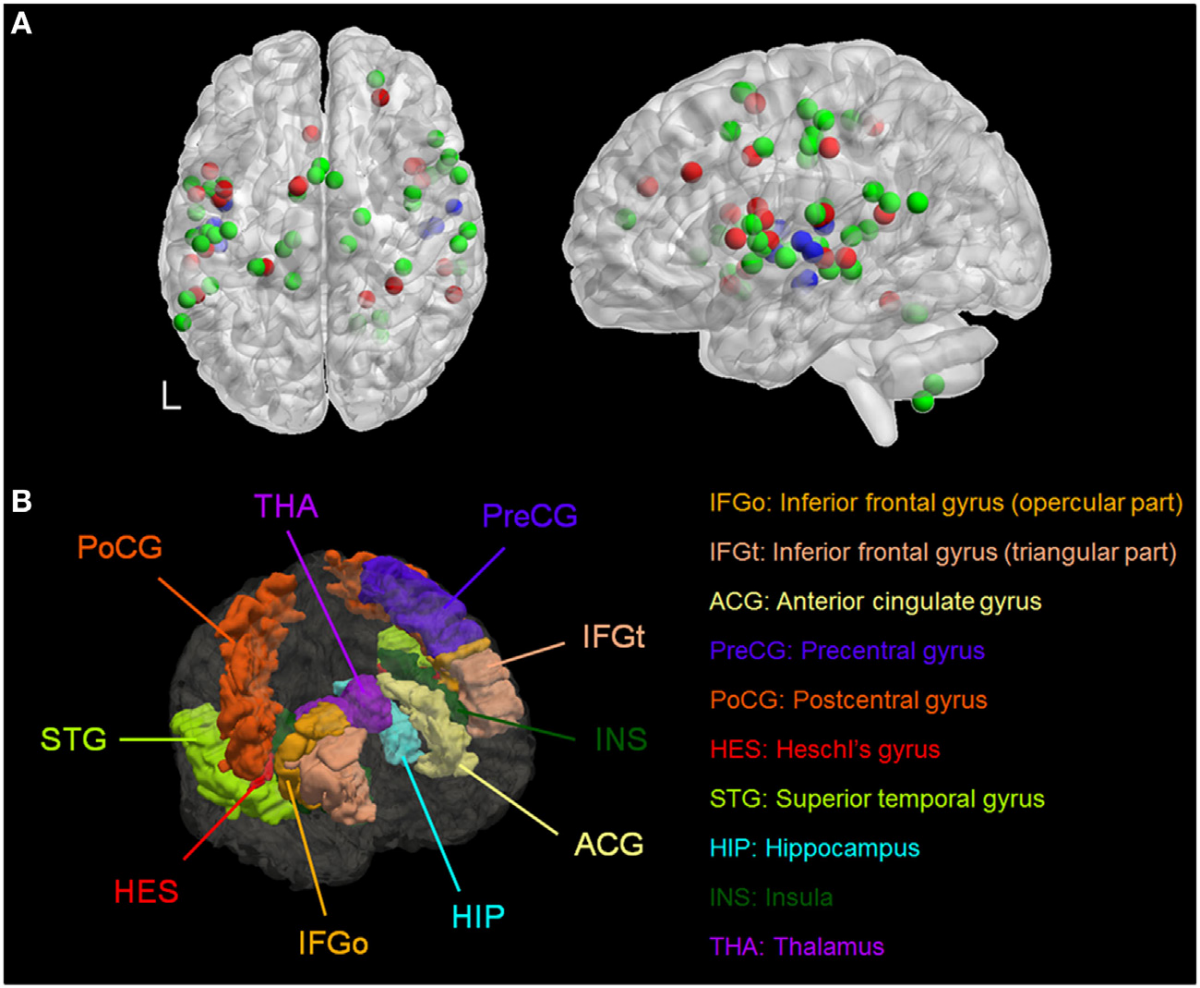
**(A)** Spatial distribution of the coordinates from meta-analyses of studies of patients with auditory verbal hallucinations (AVH) derived from Table S11 in Supplementary Material. Red = during AVH, green = during auditory or language tasks, and blue = morphometric studies. **(B)** Three-dimensional rendering of the brain regions of interest associated with AVH in a representative model. L, left.

We identified six meta-analyses that examined structural (40, 41) and functional differences in patients with AVH compared to healthy individuals (42–45). Details of the primary studies included in each meta-analysis are provided in Tables S3–S10 in Supplementary Material. Across all meta-analyses, brain regions consistently associated with AVH comprised the primary and secondary auditory cortex located in the superior temporal gyrus and in the Heschl’s gyrus; Broca’s and Wernicke’s area, respectively, located in the inferior frontal gyrus (pars opercularis and pars triangularis) and in the posterior superior temporal gyrus; the anterior cingulate gyrus; the somatosensory and motor cortices, respectively, located in the postcentral and precentral gyrus; the insula; and the hippocampus and the thalamus (Figure 1; Table S11 in Supplementary Material). Of note, most of the regions identified were BL (Table S11 in Supplementary Material). Based on these results, AVH-ROIs were prescribed in each of the five head models (Figure 1B).

### Effect of Anatomic Variation and Current Amplitude on Peak Electric Field Strength

**Figure 2:**
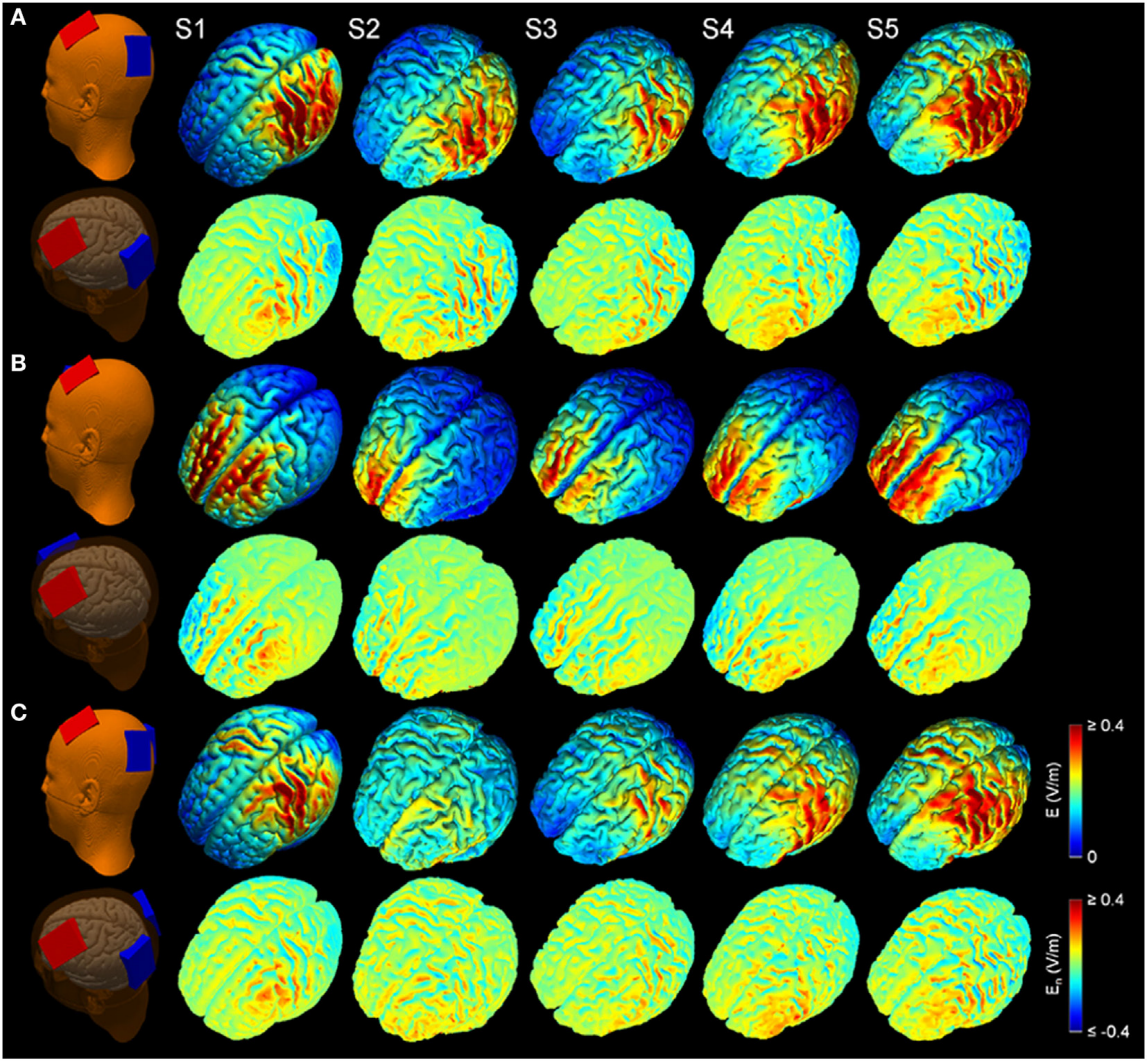
Electric field modeling of transcranial direct current stimulation (tDCS) for three montages: **(A)** left unilateral 1 × 1 montage with F3-FP1 as the anode and T3-P3 as the cathode, **(B)** bilateral prefrontal 1 × 1 montage with F3-FP1 as the anode and FP2 as the cathode, and **(C)** 2 × 1 montage with F3-FP1 as the anode and cathodes at T3-P3 and T4-P4. First column shows tDCS electrode placements representing anode (red) and cathode (blue) electrodes in the New York head model (Subject 1; S1). Rows from top to bottom show electric field magnitude (*E*) and electric field normal to the cortical surface (*E_n_*) for the models of Subjects 1–5 (S1–S5) at current amplitude of 2 mA.

**Figure 3:**
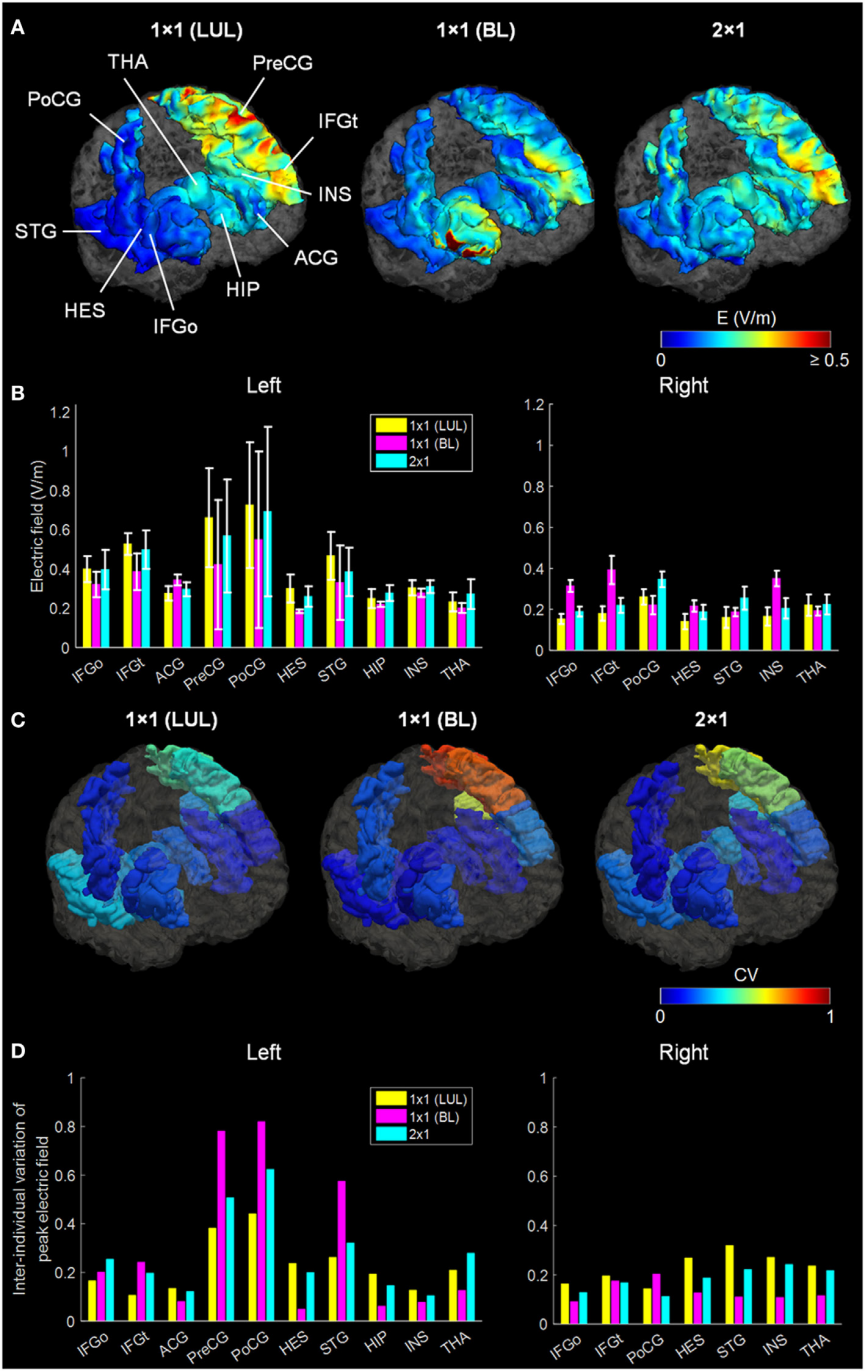
**(A)** Regional electric field strength and spatial distribution at a current of 2 mA generated by each transcranial direct current stimulation montage in a representative head model. **(B)** Peak electric field magnitude at a current of 2 mA in each auditory verbal hallucinations (AVH) region of interest (ROI). Bars show mean values, and error bars show SD across the five head models for each target AVH-ROI. **(C)** Spatial distribution of the coefficient of variation (CV) of peak electric field magnitude in each target AVH-ROI across the five head models for each montage. **(D)** CV of the peak electric field magnitude in target AVH-ROI across the five head models for each montage. LUL, left unilateral 1 × 1 montage with F3-FP1 as the anode and T3-P3 as the cathode; BL, bilateral prefrontal 1 × 1 montage with F3-FP1 as the anode and FP2 as the cathode; 2 × 1, montage with one anode at F3-FP1 and cathodes at T3-P3 and T4-P4. IFGo, inferior frontal gyrus (pars opercularis); IFGt, inferior frontal gyrus (pars triangularis); ACG, anterior cingulate gyrus; PreCG, precentral gyrus; PoCG, postcentral gyrus; HES, Heschl’s gyrus; STG, superior temporal gyrus; HIP, hippocampus; INS, insula; THA, thalamus.

Figure 2 shows the simulated LUL, BL, and 2 × 1 tDCS configurations. The spatial distributions of the electric field on the cortical surface and directional electric field normal to the cortical surface (inward and outward) are plotted for each modality in each individual. For each montage and each AVH-ROI, we estimated the mean peak electric field strength across the five head models. Figures 3A,B present the peak electric field strengths for each montage averaged across the five head models at the conventional 2 mA current amplitude. Figures 3C,D present the CV of the peak electric field strength in each AVH-ROI across the five head models for each montage. *The LUL 1* × *1 montage* predominantly induced electric fields in the left hemisphere; the inward current flow occurred mostly through the left dorsolateral prefrontal cortex while the outward current flow occurred through the left temporoparietal and occipital regions. *The BL prefrontal 1* × *1 montage* generated comparable electric field magnitude in both hemispheres; the inward and outward flow occurred mostly over the prefrontal and frontal areas. *The 2* × *1 montage* induced electric fields in the left- and in the right-sided AVH-ROIs; the inward and outflow flow occurred over the frontal cortex and the temporoparietal regions, respectively.

We found significant differences in peak electric field strength across the three electrode montages (LUL, BL, and 2 × 1) as shown in Table 1. Bonferroni-corrected pairwise comparisons showed the montages induced significantly different electric field strengths in several right-sided AVH-ROIs. Specifically, the BL montage generated higher peak electric field strengths in the right inferior frontal gyrus and insula than the other two montages (*p* < 0.001). The 2 × 1 montage produced higher peak electric field in the right postcentral gyrus than the others (*p* < 0.05).

**Table 1:** Differences in peak electric field strength for each region of interest in the auditory verbal hallucinations network across electrode montages.

Laterality	Region	Analysis of variance*F* (*p*-value)	*Post hoc* pairwise comparisons	Absolute effect size (Cohen’s*d*)		
				LUL vs BL	LUL vs 2 × 1	BL vs 2 × 1
L	Inferior frontal gyrus, pars opercularis	1.55 (0.25)	NS	1.19	0.03	0.88
L	Inferior frontal gyrus, pars triangularis	3.89 (0.051)	NS	1.84	0.36	1.17
L	Anterior cingulate gyrus	5.30 (0.02)	BL > LUL*	2.08	0.57	1.47
L	Precentral gyrus	0.85 (0.44)	NS	0.82	0.34	0.47
L	Postcentral gyrus	0.27 (0.76)	NS	0.45	0.09	0.33
L	Heschl’s gyrus	6.64 (0.01)	LUL > BL*	2.29	0.65	2.03
L	Superior temporal gyrus	1.07 (0.37)	NS	0.86	0.66	0.34
L	Hippocampus	2.97 (0.08)	NS	0.83	0.63	1.90
L	Insula	1.39 (0.28)	NS	0.81	0.17	1.14
L	Thalamus	2.16 (0.15)	NS	0.80	0.63	1.25
R	Inferior frontal gyrus, pars opercularis	52.62 (1.14e−6)	BL > LUL***; BL > 2 × 1***	5.99	1.47	4.70
R	Inferior frontal gyrus, pars triangularis	26.11 (4.25e−5)	BL > LUL***; BL > 2 × 1***	3.89	1.10	3.13
R	Postcentral gyrus	12.48 (0.001)	2 × 1 > LUL*; 2 × 1 > BL***	0.96	2.24	2.99
R	Heschl’s gyrus	6.46 (0.01)	BL > LUL*	2.30	1.29	0.91
R	Superior temporal gyrus	5.67 (0.01)	2 × 1 > LUL*	0.67	1.75	1.60
R	Insula	24.3 (6.03e−5)	BL > LUL***; BL > 2 × 1***	4.48	0.84	3.31
R	Thalamus	0.79 (0.47)	NS	0.70	0.07	0.83

Only results that survive Bonferroni correction for multiple comparisons are reported as significant; NS, non-significant; *p < 0.05; **p < 0.01; ***p < 0.001; LUL, left unilateral montage with anode over F3-FP1 and cathode over the left P3; BL, bilateral prefrontal montage with the anode over F3-FP1 and cathode over FP2; 2 × 1, one anode over the F3-FP1 and two cathodes, one over T3-P3 and the other over T4-P4; electrode placements are based on the International 10–20 system.

### Effect of Anatomic Variation and Current Amplitude on Percentage Modulated Brain Volume

**Figure 4:**
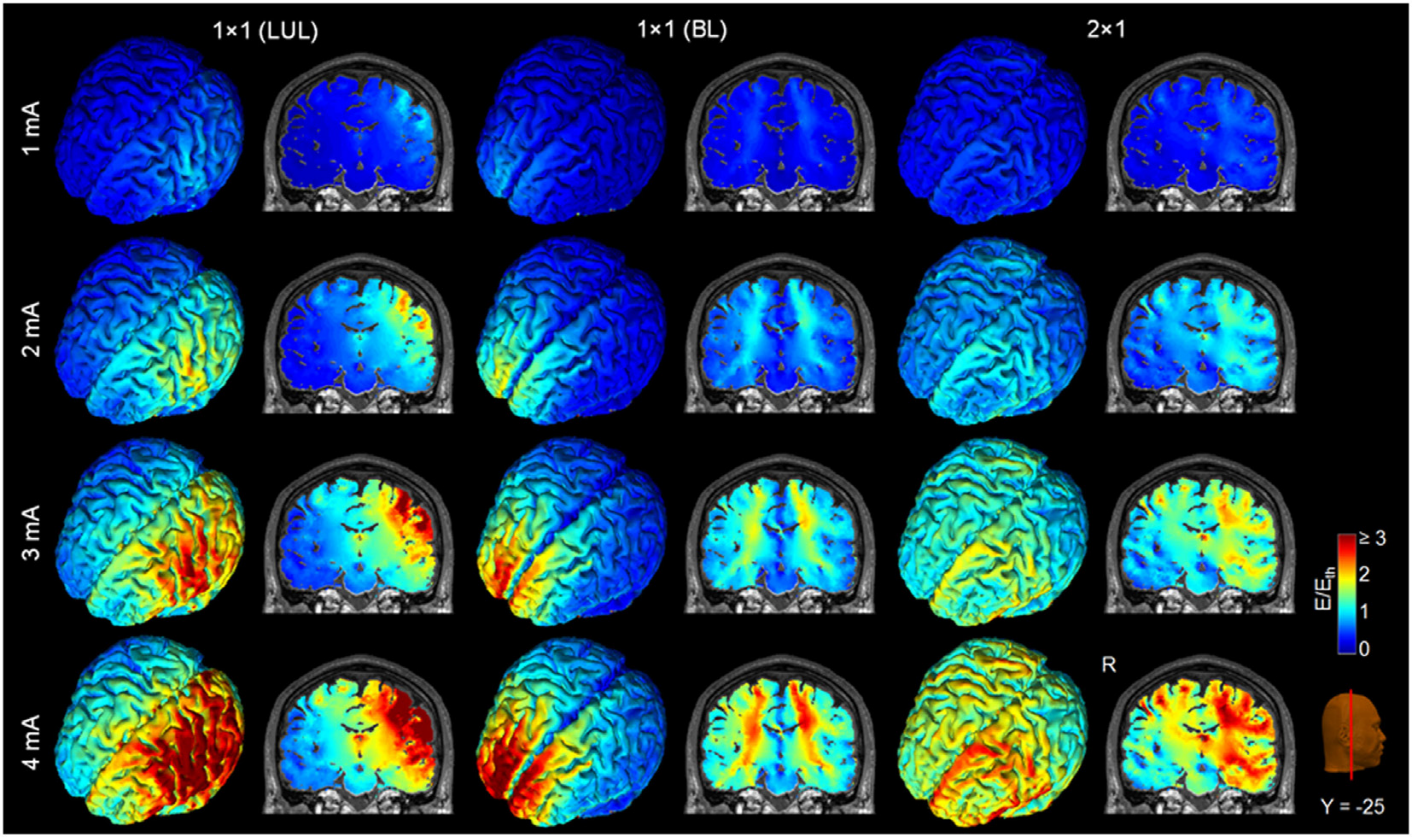
Brain regions with electric field strength above the modulation threshold (0.2 V/m) in a representative model as a function of current amplitude. Spatial distribution of the electric field shown for the cortical surface and in a representative coronal slice for current amplitudes ranging from 1 to 4 mA for each montage. LUL, left unilateral 1 × 1 montage with F3-FP1 as the anode and T3-P3 as the cathode; BL, bilateral prefrontal 1 × 1 montage with F3-FP1 as the anode and FP2 as the cathode; 2 × 1, montage with one anode at F3-FP1 and cathodes at T3-P3 and T4-P4. R, right.

**Figure 5:**
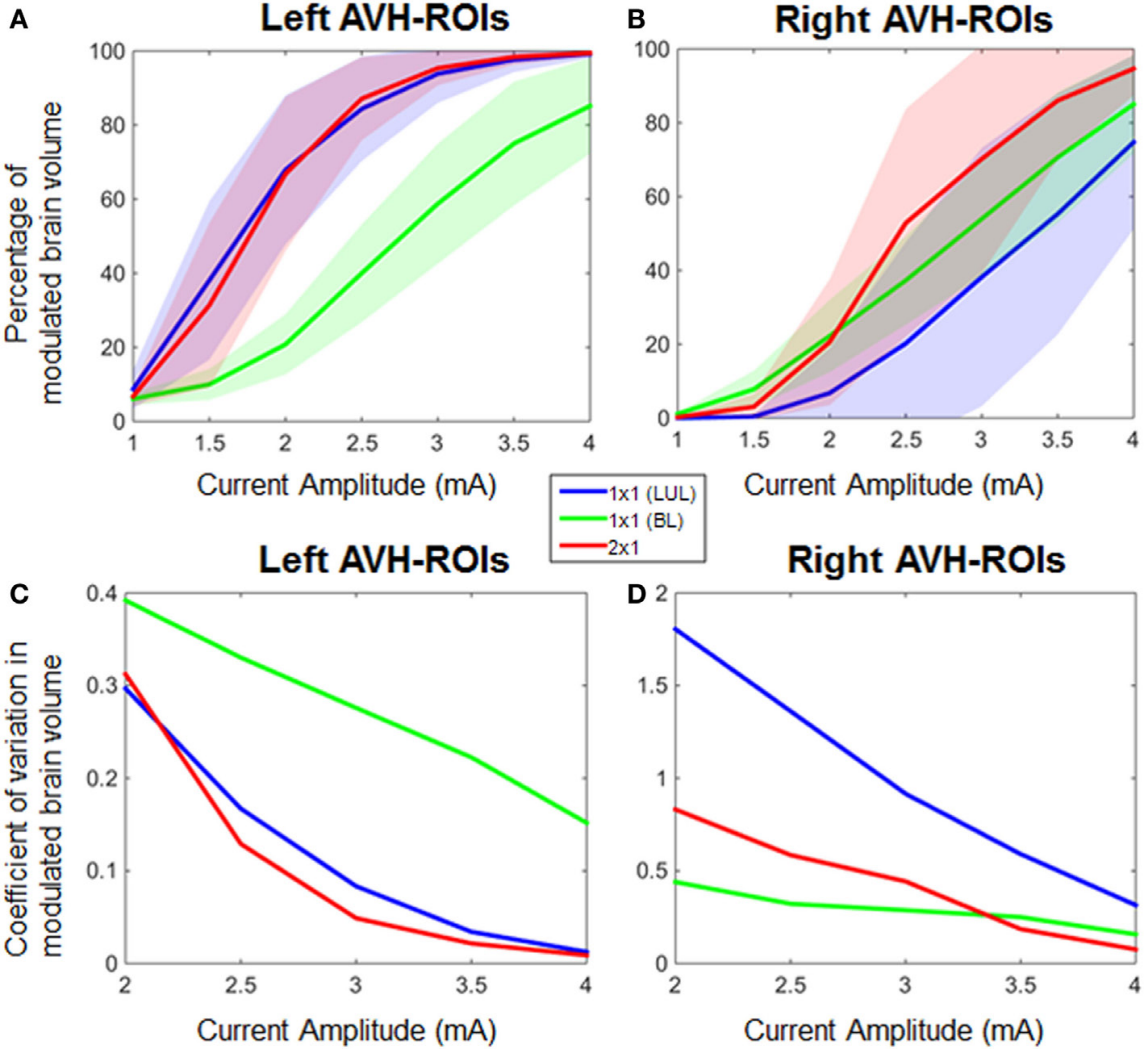
Percentage modulated volume in **(A)** left-sided and **(B)** right-sided auditory verbal hallucinations (AVH) regions of interest (ROIs) across all head models for each of the three montages as a function of current amplitude. Coefficient of variation in percentage-modulated volume in **(C)** left-sided and **(D)** right-sided AVH-ROIs across all head models for each of the three montages. LUL, left unilateral 1 × 1 montage with F3-FP1 as the anode and T3-P3 as the cathode; BL, bilateral prefrontal 1 × 1 montage with F3-FP1 as the anode and FP2 as the cathode; 2 × 1, montage with one anode at F3-FP1 and cathodes at T3-P3 and T4-P4.

Figure 4 shows the electric field maps relative to the threshold electric field (*E*/*E*_th_) for current amplitudes in the range of 1–4 mA for each montage. For each montage and each AVH-ROI in each head model, we estimated the percentage of modulated volume [i.e., percentage of the AVH network volume in which the electric field strength (*E*) was above 0.2 V/m]. We used the threshold 0.2 V/m as it is the postulated threshold for neuronal excitability (35). Figures 5A,B show the percentage modulated volume (*E* ≥ *E*_th_) for each of the three montages for the left- and right-sided AVH-ROIs for current amplitudes ranging from 1 to 4 mA averaged across the five head models. Figures 5C,D show the corresponding CV in the left- and right-sided AVH-ROIs for each montage for current amplitudes ranging from 1 to 4 mA. The corresponding data for each AVH-ROI are shown in Figures S1–S4 in Supplementary Material. Increasing the current amplitude increased modulated volumes and reduced interindividual variability, particularly for the LUL and 2 × 1 montages (Figures 4 and 5). Variability between montages reduced in general for current amplitudes above 3 mA. Nevertheless, we found significant differences in modulated volume across the three electrode montages (LUL, BL, and 2 × 1) as shown in Table 2. The modulated brain volume in the left-side AVH-ROIs was significantly different across the montages for the range of 2–4 mA at which lower modulated brain volume was produced by the BL compared to the other two montages (*p* < 0.05). In contrast, the BL generated statistically higher modulated volume in the right-side AVH-ROIs than the other two montages at low current of 1 mA (*p* < 0.001).

**Table 2:** Differences in modulated brain volume for current amplitudes in the range of 1–4 mA for the left- and right-sided regions of interest (ROIs) in the auditory verbal hallucinations network across electrode montages.

Current (mA)	Analysis of variance*F* (*p*-value)	*Post hoc* pairwise comparisons	Absolute effect size (Cohen’s*d*)		
			LUL vs BL	LUL vs 2 × 1	BL vs 2 × 1
**Left auditory verbal hallucinations (AVH)-ROIs**					
1	0.76 (0.49)	NS	0.67	0.47	0.32
1.5	3.32 (0.07)	NS	1.82	0.31	1.33
2	11.95 (0.001)	LUL > BL**; 2 × 1 > BL**	3.07	0.05	2.91
2.5	21.01 (0.0001)	LUL > BL***; 2 × 1 > BL***	3.25	0.22	3.84
3	18.57 (0.0002)	LUL > BL**; 2 × 1 > BL***	2.75	0.24	3.07
3.5	9 (0.004)	LUL > BL*; 2 × 1 > BL**	1.88	0.27	1.97
4	5.92 (0.01)	LUL > BL*; 2 × 1 > BL*	1.53	0.20	1.56
**Right AVH-ROIs**					
1	28.76 (2.6e−5)	BL > LUL***; BL > 2 × 1***	4.19	1.58	2.97
1.5	5.63 (0.01)	BL > LUL*	2.08	1.06	1.10
2	2.01 (0.17)	NS	1.40	0.93	0.12
2.5	2.16 (0.15)	NS	0.81	1.12	0.66
3	1.58 (0.24)	NS	0.58	0.97	0.66
3.5	2.17 (0.15)	NS	0.58	1.20	0.92
4	1.88 (0.19)	NS	0.53	1.14	0.90

Only results that survive Bonferroni correction for multiple comparisons are reported as significant; NS, non-significant; *p < 0.05; **p < 0.01; ***p < 0.001; LUL, left unilateral montage with anode over F3-FP1 and cathode over the left P3; BL, bilateral prefrontal montage with the anode over F3-FP1 and cathode over FP2; 2 × 1, one anode over the F3-FP1 and two cathodes, one over T3-P3 and the other over T4-P4; electrode placements are based on the International 10–20 system.

## Discussion

Here, we use AVH to illustrate the challenges in defining and assessing target engagement in the context of tDCS for psychiatric disorders and to propose a strategy for the evaluation of tDCS protocols.

There are three main challenges. First, AVH arise from dysfunction in a brain network (Figure 1) rather than a single brain region which leads to uncertainty about which regions within the AVH-related network may constitute ideal targets for tDCS (46). Moreover, commonly used montages often target left-sided AVH-ROIs while evidence from the meta-analyses (Figure 1; Table S11 in Supplementary Material) and from individual studies point to additional involvement of right-sided brain regions to the pathophysiology of AVH (47–51). Second, there is inter-individual variability in brain structure and function in general (52) and in the AVH-related network in particular (50, 51). Individual differences in the spatial distribution of tDCS-induced electric fields may impact on the engagement of AVH-related regions and hence clinical response. Third, there is no behavioral, cognitive or neurophysiological measure that can be used to assess AVH-related neural changes during or following a single tDCS session, in contrast to electroconvulsive therapy (53) and repetitive transcranial magnetic stimulation (54) where seizure and motor excitability threshold can be, respectively, used as a threshold for titrating treatment.

In response to these uncertainties, we tested whether computational modeling of electric field measures induced by tDCS can be meaningfully used to assess the engagement of the AVH network. We mined the available neuroimaging literature to define target ROIs implicated in AVH (Figure 1) and used computational modeling approach to assess the engagement of these ROIs in five head models using the three different tDCS montages (Figure 2). The search space for optimal electrode placement in the treatment of AVH is too vast for empirical evaluation of brute force. Taking a pragmatic approach, we considered the LUL and BL prefrontal montages as these are most commonly used in the literature (Tables S1 and S2 in Supplementary Material). We also evaluated an experimental 2 × 1 montage, because of its theoretical potential to provide better target engagement of right-sided target AVH-ROIs. We used two measures of target engagement, specifically peak electric field magnitude in each AVH-ROI and modulated brain volume of the AVH-network.

In terms of peak electrical field strength, the three montages differed only in a few right-sided AVH-ROIs. Specifically, the BL montage generated higher peak electric field strengths in the right inferior frontal gyrus and insula, while the 2 × 1 montage produced higher peak electric field in the right postcentral gyrus. Both of these regions have been associated with increased AVH frequency in patients who experience persistent AVH (44). The strength of the tDCS-induced electric field is a conventional measure of assessing the spatial distribution of the electric fields generated by different electrode configurations. However, its functional significance in terms of clinical efficacy has not been tested and is currently unknown. If we assume that peak electric field strength is associated with clinical efficacy, the current results suggest that the commonly used LUL montage is perhaps the least advantageous if one is interested in electrode montages that have the potential to modulate right-sided AVH-ROIs.

The three montages also differed in terms of modulated volume. In this case, however, differences were noted for the left-sided AVH network where both the LUL and 2 × 1 montages produced higher modulated volumes than the BL for currents ranging from 2 to 4 mA. As expected, the BL montage produced the highest focality within frontal regions due to the proximity of the electrodes (21). Consistent with previous findings (21), increasing the current amplitude increased modulated volume within AVH network. For the threshold-based modulated volume measure, variability between montages reduced in general for current amplitudes above 3 mA. Reduced interindividual variation at higher currents resulted from increased modulated volume within AVH network across multiple realistic head models. This is important for clinical applications, since it implies that increasing the current amplitude may be one possible option to overcoming anatomical variability and uncertainty about optimal electrode placement and potentially improve the efficacy of tDCS for AVH.

This study provides a computational evidence for two different measures of target engagement that behave differently across different montages. There are other parameters in tDCS protocols involving duration of tDCS session, interval of administration, and length of trial (55) that may contribute to efficacy but could not be examined here. We did not address the issue of tolerability for higher current amplitudes although the excellent tolerability of tDCS allows optimism (18, 31, 56). Nevertheless, we have provided a road map for the development of target engagement measures for tDCS that is based on neuroimaging evidence regarding the target networks and accommodates uncertainty about patient-specific abnormalities. This translational approach not only combines advances in computational modeling with knowledge gained from behavioral and functional neuroimaging studies regarding target definition and target engagement but also highlights knowledge gaps and points to avenues for future research.

Generally, the principled computational approach developed here could inform target engagement across tDCS application. Prior computational models typically considered electric field magnitude (or normal direction) in a single ROI. We show that consideration of multiple nodes in a functional network (based on imaging and trial meta-analysis) forces decisions on region stimulation and sparring that then suggest divergent optimal montages. The approach outlined here is particularly relevant for study designs that adopt the Research Domain Criteria (RDoC) framework.^2^ The RDoC initiative specifies neural circuits that may be theoretically and empirically linked to clinical symptoms and cognitive constructs. This framework therefore can be used to aid in the specification of target networks in future tDCS studies in neuropsychiatry. Moreover, considering a simple linear electric field (magnitude or normal) vs non-linear measures of ROI influence (such as our threshold-based volume measures) leads to different conclusion on the value of current intensity and best montage. As such, tDCS interventions rationalized based on computational modeling should be explicit about the underlying assumptions regarding network (dys)function and side effects (i.e., selection of target and avoid ROIs), biophysics of stimulation (i.e., measure of ROI engagement), and goals (e.g., reduce interindividual variation).

Our approach also addresses issues relevant to personalized psychiatry because, as we have shown, individual variations in electrical field distribution in tDCS can be overcome by protocol modifications such as increasing the current amplitude to 4 mA.

## Ethics Statement

This study makes a secondary use of experimental data already published (20–22). All subjects gave written informed consent in accordance with the Declaration of Helsinki.

## Author Contributions

WL, NK, and SF conceived and designed the study. WL performed the analysis and computation. NK and SF performed the literature survey and meta-analyses. MB provided three individual head models and helped the data interpretation. All the authors wrote the manuscript, analyzed the data, and reviewed the manuscript.

## Conflict of Interest Statement

WL, NK, and SF declare no competing financial interest. The City University of New York has patents on brain stimulation with MB as an inventor. MB has equity in Soterix Medical Inc.

## Footnotes

^1^http://www.brainmap.org/icbm2tal/.

^2^https://www.nimh.nih.gov/research-priorities/rdoc/index.shtml.
