# Supplementary material for: A Computational Assessment of Target Engagement in the Treatment of Auditory Hallucinations with Transcranial Direct Current Stimulation
Source: Front Psychiatry. 2018 Feb 22;9:48. doi: 10.3389/fpsyt.2018.00048 (PMC5826940; doi:10.3389/fpsyt.2018.00048)
Supplement: Supplementary file 1 [file Data_Sheet_1.docx]

**Supplementary Material**

**A computational assessment of target engagement in the treatment of auditory hallucinations with transcranial direct current stimulation**

**1. Clinical efficacy and tolerability of tDCS in AVH in schizophrenia spectrum disorders**

Studies examining the efficacy and tolerability of tDCS on auditory verbal hallucinations (AVH) in schizophrenia are Supplementary Tables S1 and S2. The former summarizes open label studies and case series and the later summarizes the randomised controlled trials (RCT). There was little variation between studies with regards to patient selection and assessment. Patients selected had persistent AVH despite adequate antipsychotic treatment. The severity of AVH was most commonly assessed using the Auditory Hallucinations Rating subscale (AHRS) of the Psychotic Symptoms Rating Scales (PSYRATS) (Haddock et al., 1999) or the relevant score on the Positive Subscale of the Positive and Negative Syndrome Scale (Kay et al., 1987).

| **Table S1. Studies using transcranial direct current stimulation (tDCS) for the treatment of auditory verbal hallucinations** | | | | | | | | | | |
| --- | --- | --- | --- | --- | --- | --- | --- | --- | --- | --- |
| **Author** | | **Year** | **Sample**  **(n)** | **Baseline features**  **(mean, SD)** | **Sessions** | **Electrode size** | **Current (mA)** | **Anode^1^** | **Cathode^1^** | **Results** |
| **Safety and Tolerability Studies** | | | | | | | | | | |
| Mattai et al | | 2011 | 8 | **Sample: 5 male, 3 female**  Age 15.31  Symptom ratings not reported | 10 total – once daily for 5 consecutive days, weekend break, 5 consecutive days; each session lasted 20 min | 5x5cm | 2 | FP1 and FP2 | No cathode | Tolerability study; Clinical efficacy not assessed  Tingling and fatigue |
|  |  |  | 5 | **Sample: 1 male, 4 female**  Age 15.5  Symptom ratings not reported | 10 total – once daily for 5 consecutive days, weekend break, 5 consecutive days: each session lasted 20 min | 5x5cm | 2 | No anode | T3 bilateral | Tolerability study; Clinical efficacy not assessed  Tingling and fatigue |
| **Open Label Trials** | | | | | | | | | | |
| Agarwal et al | | 2016 | 36 | **Sample: 15 male, 11 female**  Age: 33.27 (12.6)  AHRS: 31.2 (4.7) | 10 total – twice daily for 5 consecutive days; each session lasted 20 min | 5x7cm | 2 | F3-FP1 | T3-P3 | Significant effect of tDCS based on the AHRS at end of treatment period; effect size *d*=1.25; Patients on first generation antipsychotics benefited less than those on newer antipsychotic agents  Tingling |
| Shivakumar et al | | 2015 | 23 | **Sample: 10 male, 13 female**  Age: 33.4 (12.7)  PSYRATS –AH subscale items: Frequency, duration, disruption all **>2** | 10 total – twice daily for 5 consecutive days; each session lasted 20 min | 5x7cm | 2 | F3-FP1 | T3-P3 | Repeated measures ANOVA with COMT Val/Met genotype between subjects showed significant reduction in AHS scores after tDCS in both groups.  No adverse events reported |
| Subramaniam et al | | 2015 | 13 | **Sample: 4 male, 9 female**  Age = 29.6 (8.1)  AHRS: 30.6 (3.6) | 10 total – twice daily for 5 consecutive days; each session lasted 20 min | 5x7cm | 2 | F3-FP1 | T3-P3 | Significant effect of tDCS based on the AHRS at end of treatment period; effect size *d*=1.88  No adverse events reported |
| Nawani et al | | 2014a | 5 | **Sample: 2 male, 3 female**  Age 33.2 (19.3)  AHRS: 33 (4.9) | 10 total – twice daily for 5 consecutive days; each session lasted 20 min | 5x7cm | 2 | F3-FP1 | T3-P3 | Significant effect of tDCS based on the AHRS at end of treatment period; effect size *d*=1.57  Tingling |
| Bose et al | | 2014 | 21 | **Sample: 9 male, 12 female**  Age: 33.1 (12.8)  AHRS: 32.1 (4.1) | 10 total – twice daily for 5 consecutive days; each session lasted 20 min | 5x7cm | 2 | F3-FP1 | T3-P3 | Significant effect of tDCS based on the AHRS at end of treatment period; effect size *d*=1.24  No adverse events reported |
| **Case Reports** | | | | | | | | | | |
| Praharaj et al | 2015 | | 1 | Male; Age: 49 | 5 total – once daily for 5 consecutive days | 5x5cm | 2 | F3 | T3-P3 | More than 90% reduction in both the frequency and duration of auditory  hallucinations from baseline levels  No adverse events reported |
| Shenoy et al | 2015 | | 1 | Female; Age: 25 | 10 total – twice daily for 5 consecutive days; each session lasted 20 min | 5x7cm | 2 | F3-FP1 | T3-P3 | AHRS reduced by 24.1% at 1 week,93.1% by 1 month.  No adverse events reported |
| Bose et al | 2015 | | 1 | Female; Age: 37 | 20 total – twice daily for 5 consecutive days, weekend break, twice daily for another 5 consecutive days each session lasted 20 min | 5x7cm | 2 | F4-FP2 | T4-P4 | AHRS reduced by 31.4% after failing to respond to prior treatment with left unilateral tDCS montage  No adverse events reported |
| Nawani et al | 2014b | | 1 | Male; Age: 31 | 10 total – twice daily for 5 consecutive days; each session lasted 20 min | 5x7cm | 2 | F3 | T3-P3 | Unspecified improvement in AVH based on AHRS  No adverse events reported |
| Narayanaswamy et al | 2014 | | 1 | Female; Age: 22 | 10 total – twice daily for 5 consecutive days; each session lasted 20 min | 5x7cm | 2 | F3-FP1 | T3-P3 | Unspecified improvement in AVH  No adverse events reported |
| Shivakumar et al | 2014 | | 1 | Female; Age: 42 | 10 total – twice daily for 5 consecutive days; followed by booster sessions (2 sessions on a single day) over 3 monthly intervals; each session lasted 20 min | 5x7cm | 2 | F3-FP1 | T3-P3 | On demand tDCS for relapse of AVH in patient who remitted fully after initial treatment  No adverse events reported |
| Andrade | 2013b | | 1 | Female; Age: 24 | Once daily for > 6 months; each session lasted 20 min | 5x5cm | 1 | F3 | T3-P3 | Sustained unspecified reduction in AVH severity  Tingling |
| Rakesh et al | 2013 | | 1 | Male; Age: 24 | 10 total – twice daily for 5 consecutive days: each session lasted 20 min | 5x7cm | 2 | AF3 | T3-P3 | AHRS score 0 at the end of treatment period  No adverse events reported |
| Shiozawa et al | 2013 | | 1 | Male; Age: 35 | 20 total – Once daily for 10 consecutive days, 5 rest days, once daily for 10 consecutive days | Unspecified | 2 | F3 | Oz first 10 sessions, T3-P3 second 10 sessions | Reduction (20 %) in the AHRS score at the end of treatment period; improvement maintained at 4-week follow-up  No adverse events reported |
| Shivakumar et al | 2013 | | 1 | Female; Age 28 | 10 total – twice daily for 5 consecutive days; each session lasted 20 min | 5x7cm | 2 | F3-FP1 | T3-P3 | AHRS score 0 at the end of treatment period  No adverse events reported |
| Homan et al | 2011 | | 1 | Male; Age: 44 | 10 total – twice daily for 5 consecutive days; each session lasted 15 min | 5x7cm | 1 | Frontal unspecified | Temporo-patietal  unspecified | Reduction (60 %) in the Hallucination Change Scale Score at end of treatment period; improvement maintained at 6-week follow-up  No adverse events reported |
| 1=Electrodes placements as specified by the international 10-20 electrode placement system; 2=Overlapping patient samples; 3= the study involved children and adolescents with childhood onset schizophrenia; AHRS=Auditory Hallucination Rating Scale; AVH= Auditory verbal hallucinations PANSS=Positive and Negative Syndrome Scale; continuous variables shown as mean (standard deviation); age in years. | | | | | | | | | | |

| **Table S2. Details of stimulation parameters of the randomized clinical trials of tDCS in auditory hallucinations** | | | | | | | | | | |
| --- | --- | --- | --- | --- | --- | --- | --- | --- | --- | --- |
| **Study**  **(First Author, Year)** | **Number of patients in the active condition** | **Number of patients in the sham condition** | **Anode placement** | **Cathode placement** | **Current amplitude**  **(mA)** | **Electrode surface area**  **(cm^2^)** | **Number of sessions** | **Frequency of treatment** | **Outcome measures included in meta-analysis** | **Other outcome measures** |
| Brunelin 2012 | 15 | 15 | F3/Fp1 | T3/P3 | 2 | 35cm^2^ | 10 | Twice daily | AHRS, PANSS |  |
| Fitzgerald 2014 | 24 | 24 | F3  or  F3/4 | TP3  or  TP3/4 | 2 | 35cm^2^ | 15 | Once daily | PANSS | CDS,  SANS |
| Frohlich 2016 | 13 | 15 | F3/Fp1 | T3/P3 | 2 | 35cm^2^ | 5 | Once daily | AHRS, PANSS |  |
| Mondino 2014 | 11 | 12 | F3/Fp1 | T3/P3 | 2 | 35cm^2^ | 10 | Twice daily | AHRS, PANSS |  |
| Smith 2015 | 17 | 16 | F3 | Fp2 | 2 | 2in ^2^ | 5 | Once daily | AHRS, PANSS | MCCB, PSYRATS |
| AHRS=Auditory Hallucinations Rating Scale; CDS=Calgary Depression Scale; MCCB=MATRICS Consensus Cognitive Battery; PANSS=Positive and Negative Syndrome Scale; PSYRATS=Psychotic Symptoms Rating Scale; SANS=Scale for the Assessment of Negative Symptoms; tDCS=Transcranial Direct Current Stimulation; Electrode placement according to the International 10-20 system. | | | | | | | | | | |

**Tables S3-10: Details of primary studies included in meta-analyses of brain abnormalities related to auditory verbal hallucinations**

| **Table S3. Details of studies of state related activation during auditory verbal hallucinations included in the meta-analysis of Jardri et al (2011)** | | | | |
| --- | --- | --- | --- | --- |
| **Studies included** | **Modality** | **Patients n=69**  **Healthy controls n=8** | **Definition of AVH occurring during scan** | **Results of the meta-analysis**  **(x, y, z)** |
| Copolov et al., 2003 | PET | Schizophrenia=7  Schizoaffective=1  Healthy controls=8 | Button press during AVH | **Cluster A**  Broca’s area  (–48, 10, 7)  Anterior insula  (–42, 0, 6)  Precentral gyrus  (–54, 0, 14)  **Cluster B**  Hippocampus/parahippocampal gyrus  (–24, –32, –4)  **Cluster C**  Anterior insula  (44, 6, –4)  Frontal operculum  (42, 12, –10)  **Cluster D**  Superior and middle temporal gyri  (–54, –44, 16)  **Cluster E**  Supramarginal gyrus  (–52, –20, 15) |
| Dierks et al., 1999 | fMRI | Schizophrenia=3  No healthy controls | Button press during AVH |  |
| Jardri et al., 2007 | fMRI | Childhood onset schizophrenia=1  No control subjects | Interview post scan |  |
| Jardri et al., 2009 | fMRI | Schizophrenia=1  No healthy controls | Button press during AVH |  |
| Jardri et al., 2009 | fMRI | First episode schizophrenia=15  No healthy controls | Interview post scan |  |
| Lennox et al., 1999 | fMRI | Schizophrenia=4  No healthy controls | Button press during AVH |  |
| Shergill et al., 2000 | fMRI | Schizophrenia =6  No healthy controls | Random sampling and button press during AVH |  |
| Shergill et al., 2001 | fMRI | Schizophrenia =1  No healthy controls | Random sampling |  |
| Silbersweig et al., 1995 | PET | Schizophrenia=5  Non-clinical diagnosis with auditory & visual hallucinations=1  No healthy controls | Button press during AVH |  |
| Sommer et al., 2008 | fMRI | Schizophrenia=18  Schizoaffective disorder=3  Unspecified psychotic disorder=3  No healthy controls | Button press during AVH |  |
| AVH=auditory verbal hallucinations; fMRI= functional magnetic resonance imaging; PET=Positron Emission Tomography; x=sagittal; y=coronal; z=axial; results presented as reported by Jardri et al (2011). | | | | |

| **Table S4. Details of studies of state related activation during auditory verbal hallucinations included in the meta-analysis of Kühn and Gallinat (2012)** | | | | |
| --- | --- | --- | --- | --- |
| **Included Studies** | **Modality** | **Patients n=71**  **Healthy controls n=23** | **Definition of AVH occurring during scan** | **Results of the meta-analysis**  **(x, y, z)** |
| Copolov et al., 2003 | PET | Schizophrenia=7  Schizoaffective =1  Healthy controls=8 | Button press during AVH | Inferior frontal gyrus  (-42, 2, 18)  Anterior cingulate gyrus  (-4, 26, 31) and (-9, 4, 37)  Superior temporal gyrus  (-44, -22, 0)    Middle temporal gyrus  (-56, -30, 0)  Premotor cortex  (-10, 3, 56) |
| Diederen et al., 2010 | fMRI | Schizophrenia=16  Schizoaffective=2  Schizophreniform=1  Unspecified psychotic disorder=5  Healthy controls= 5 | Button press during AVH |  |
| Dierks et al., 1999 | fMRI | Schizophrenia=3  No healthy controls | Button press during AVH |  |
| Hoffman et al., 2008 | fMRI | Schizophrenia or schizoaffective disorder=6  No healthy controls | Button press during AVH |  |
| Lennox et al., 2000 | fMRI | Schizophrenia=4  No healthy controls | Button press during AVH |  |
| Raij et al., 2009 | fMRI | Schizophrenia=7  Schizoaffective disorder=4  No healthy controls | Button press during AVH |  |
| Shergill et al., 2000 | fMRI | Schizophrenia=6  No healthy controls | Random sampling |  |
| Shergill et al., 2001 | fMRI | Schizophrenia=1  No healthy controls | Random sampling |  |
| Shergill et al., 2004 | fMRI | Schizophrenia=2  No healthy controls | Button press during AVH |  |
| Silbersweig et al., 1995 | PET | Non-clinical diagnosis with auditory & visual hallucinations=1  No healthy controls | Button press during AVH |  |
| AVH=auditory verbal hallucinations; fMRI=functional magnetic resonance imaging; PET=Positron Emission Tomography; x=sagittal; y=coronal; z=axial; results presented as reported by Kühn and Gallinat (2012). | | | | |

| **Table S5.**  **Details of studies of state related activation during auditory verbal hallucination included in the meta-analysis of Kompus et al (2011)** | | | | |
| --- | --- | --- | --- | --- |
| **Studies included** | **Modality** | **Patients n=103**  **No healthy controls** | **Definition of AVH occurring during scan** | **Results of the meta-analysis**  **(x, y, z)** |
| Barkus et al., 2007 | fMRI | Hallucination prone healthy adults=8 | Signal detection in white noise paradigm | Superior frontal gyrus  (26, 42, 26)  Inferior frontal gyrus  (40, 12, 16)  Superior temporal gyrus  (−52, −22, 16) and (58, −44, 14)  Middle temporal gyrus  (54, −32, −4)  Postcentral gyrus  (−50, −24, 40)  Inferior parietal lobule  (32, −40, 48)  Insula (−44, −2, 6)  Hippocampus  (−24, −32, −4)  Cerebellum  (20, −46, −16) |
| Copolov et al., 2003 | PET | Schizophrenia =7  Schizoaffective =1 | Button press during AVH |  |
| Diederen et al., 2010 | fMRI | Schizophrenia=16  Schizoaffective=2  Schizophreniform=1  Unspecified psychotic disorder=5 | Button press during AVH |  |
| Dierks et al., 1999 | fMRI | Schizophrenia=3 | Button press during AVH |  |
| Lennox et al., 1999 | fMRI | Schizophrenia=4 | Button press during AVH |  |
| Linden et al., 2011 | fMRI | Nonclinical hallucinations=7 | Button press during AVH |  |
| Raij et al., 2009 | fMRI | Schizophrenia=7  Schizoaffective disorder=4 | Button press during AVH |  |
| Shergill et al., 2000 | fMRI | Schizophrenia=6  No control subjects | Random sampling |  |
| Shergill et al., 2001 | fMRI | Schizophrenia=1 | Random sampling |  |
| Shergill et al., 2004 | fMRI | Schizophrenia=2 | Button press during AVH |  |
| Silbersweig et al., 1995 | PET | Schizophrenia=5 | Button press during AVH |  |
| Sommer et al., 2008 | fMRI | Schizophrenia=18  Schizoaffective disorder=3  Unspecified psychotic disorder=3 | Button press during AVH |  |
| AVH=auditory verbal hallucinations; fMRI=functional magnetic resonance imaging; PET=Positron Emission Tomography; x=sagittal; y=coronal; z=axial; results presented as reported by Kompus et al (2011). | | | | |

| **Table S6. Details of task related studies in individuals with a history of auditory verbal hallucinations included in the meta-analysis of Kühn and Gallinat (2012)** | | | | |
| --- | --- | --- | --- | --- |
| **Studies included** | **Modality** | **Patients n=120**  **Healthy controls n=69** | **Task** | **Results of the meta-analysis**  **(x, y, z)** |
| Allen et al., 2007 | fMRI | Schizophrenia with hallucinations=10  Schizophrenia without hallucinations=10  Healthy controls =11 | Listening to speech: self vs non self | Inferior frontal gyrus  (-48, 2, 6)  Parietal operculum  (-55, -19, 16)  Postcentral gyrus  (-49, -17, 41) and (36, -32, 50) |
| Copolov et al., 2003 | PET | Schizophrenia =7  Schizoaffective =1  Healthy controls =8 | Listening to speech |  |
| McGuire et al., 1995 | PET | Schizophrenia with hallucinations= 6  Schizophrenia without hallucinations=6  Healthy controls= 6 | Imagining speech of another person |  |
| Shergill et al., 2001 | fMRI | Schizophrenia with hallucinations=8  Healthy controls=8 | Inner speech |  |
| Simons et al., 2010 | fMRI | Schizophrenia with hallucinations=15  Healthy controls=12 | Inner speech vs listening |  |
| Stephane et al., 2006 | fMRI | Schizophrenia with hallucinations=8  Schizophrenia without hallucinations=10  Healthy controls=11 | Reading nouns vs looking at nouns |  |
| Zhang et al., 2008* | fMRI | Schizophrenia with hallucinations=13  Healthy controls=13 | Judge direction of speech; Voice recognition |  |
| AVH=auditory verbal hallucinations; fMRI=functional magnetic resonance imaging; PET=Positron Emission Tomography; x=sagittal; y=coronal; z=axial; results presented as reported by Kühn and Gallinat (2012); * the study also included patients without AVH not considered here. | | | | |

| **Table S7. Details of task related studies in individuals with a history of auditory verbal hallucinations included in the meta-analysis of Kompus et al (2011)** | | | | |
| --- | --- | --- | --- | --- |
| **Studies included** | **Modality** | **Patients n=229**  **Healthy controls n=170** | **Study design** | **Results of the meta-analysis**  **(x, y, z)** |
| Braus et al., 2002 | fMRI | Schizophrenia with hallucinations=12  Healthy controls=11 | Auditory stimulation task (drumbeats) | Superior frontal gyrus  (24, 50, 14)  Superior temporal gyrus  (−54, −8, 0)  Anterior cingulate gyrus  (−10, 0, 40)  Thalamus  (12, −22, 18)  Hippocampus  (−12, −38, 10) |
| Ganguli et al., 1997 | PET | Schizophrenia with hallucinations=8  Healthy controls=8 | Verbal free-recall supra-span memory task |  |
| Kang et al., 2009 | fMRI | Schizophrenia with hallucinations=14  Schizophrenia without hallucinations=14  Healthy controls=28 | Processing of laughing/crying sounds |  |
| Koeda et al., 2006 | fMRI | Schizophrenia with hallucinations=14  Healthy controls=14 | Auditory stimulation  (sentences) |  |
| Kumari et al., 2010 | fMRI | Schizophrenia with hallucinations= 63  Healthy controls=20 | Monitoring of self and externally generated speech |  |
| Mitchell et al., 2004 | fMRI | Schizophrenia with hallucinations=12  Bipolar disorder with hallucinations=11  Healthy controls=13 | Auditory stimulation (emotional sentences) |  |
| Morey et al., 2008 | fMRI | Schizophrenia with hallucinations=26  Healthy controls=17 | Auditory stimulation (tones) |  |
| Ngan et al., 2003 | fMRI | Schizophrenia with hallucinations n= 14  Healthy controls=29 | Audio detection task (complex non-speech sounds) |  |
| Ojeda et al., 2002 | PET | Schizophrenia with hallucinations n=11  Healthy controls=10 | Mental counting task during auditory stimulation |  |
| Simons et al., 2010 | fMRI | Schizophrenia with hallucinations n=15  Healthy controls=12 | Auditory stimulation (sentences)/imagined inner speech |  |
| Woodruff et al., 1997 | fMRI | Schizophrenia with hallucinations=8  Schizophrenia without hallucinations=7  Healthy controls=8 | Auditory stimulation (sentences) |  |
| AVH=auditory verbal hallucinations; fMRI=functional magnetic resonance imaging; PET=Positron Emission Tomography; x=sagittal; y=coronal; z=axial; results presented as reported by Kompus et al (2011). | | | | |

| **Table S8.**  **Details of trait related morphometric studies of patients with auditory verbal hallucinations included in the meta-analysis of Modinos et al (2013)** | | | | |
| --- | --- | --- | --- | --- |
| **Studies included** | **Modality** | **Patients n=406**  **Healthy controls n=146** | **Study design** | **Results of the meta-analysis**  **(x, y, z)** |
| van Tol et al., 2013 | MRI | Schizophrenia with hallucinations=31  Schizophrenia without hallucinations=20  Healthy controls=51 | VBM | Superior temporal gyrus  (-52, -18, 2) and (46, -16, -8)  Rolandic operculum  (-44, -22, 12)  Heschl’s gyrus  (-46, -14, 6) and (50, -14, 6)  Middle temporal gyrus  (50, -14, -10) |
| Nenadic et al., 2010 | MRI | Schizophrenia with hallucinations=38  Schizophrenia without hallucinations=61  No healthy controls | VBM |  |
| Modinos et al., 2009 | MRI | Schizophrenia with hallucinations=26  No healthy controls | VBM |  |
| García-Martí et al., 2008 | MRI | Schizophrenia with hallucinations=18  Healthy controls=19 | VBM |  |
| O’Daly et al., 2007 | MRI | Schizophrenia with hallucinations=28  Healthy controls=32 | VBM |  |
| Neckelmann et al., 2006 | MRI | Schizophrenia with hallucinations=12  Healthy controls=12 | VBM |  |
| Plaze et al., 2006 | MRI | Schizophrenia with hallucinations=15  No healthy controls | VBM |  |
| Gaser et al., 2004 | MRI | Schizophrenia with hallucinations=29  Schizophrenia without hallucinations=56  No healthy controls | VBM |  |
| Shapleske et al., 2002 | MRI | Schizophrenia with hallucinations=41  Schizophrenia without hallucinations= 31  Healthy controls=32 | VBM |  |
| MRI=magnetic resonance imaging; x=sagittal; y=coronal; z=axial; results presented as reported by Modinos et al (2013). | | | | |

| **Table S9. Details of trait related morphometric studies of patients with auditory verbal hallucinations included in the meta-analysis of Palaniyappan et al (2012)** | | | | |
| --- | --- | --- | --- | --- |
| **Studies included** | **Modality** | **Patients n=340**  **Healthy controls n=95** | **Study design** | **Results of the meta-analysis**  **(x, y, z)** |
| García-Martí et al., 2008 | MRI | Schizophrenia with hallucinations=18  Healthy Controls=19 | VBM | Insula  (−42, −4, 2)  Superior temporal gyrus  (58, −6, 10) |
| Gaser et al., 2004 | MRI | Schizophrenia with hallucinations=29  Schizophrenia without hallucinations=56  No healthy controls | VBM |  |
| Modinos et al., 2009 | MRI | Schizophrenia with hallucinations=26  No healthy controls | VBM |  |
| Neckelmann et al., 2006 | MRI | Schizophrenia with hallucinations=12  Healthy Controls=12 | VBM |  |
| Nenadic et al., 2010 | MRI | Schizophrenia with hallucinations=38  Schizophrenia without hallucinations=61  No healthy controls | VBM |  |
| O’Daly et al., 2007 | MRI | Schizophrenia with hallucinations=28  Healthy Controls=32 | VBM |  |
| Shapleske et al., 2002 | MRI | Schizophrenia with hallucinations=41  Schizophrenia without hallucinations=31  Healthy Controls=32 | VBM |  |
| MRI=magnetic resonance imaging; x=sagittal; y=coronal; z=axial; results presented as reported by Palaniyappan et al (2012). | | | | |

| **Table S10. Details of studies of state related activation during auditory verbal hallucination included in the meta-analysis of Zmigrod et al (2016)** | | | | |
| --- | --- | --- | --- | --- |
| **Studies included** | **Modality** | **Patients n=165**  **Healthy controls n=28** | **Study design** | **Results of the meta-analysis (x, y, z)** |
| Blom et al., 2011 | fMRI | Alice in Wonderland syndrome=1  No healthy controls | Semi structured interview | Postcentral Gyrus  (-46, -18, 44)  (-52, -22, 50)  (-40, -16, 54)  (60, -22, 44)  (64, -16, 36)  Superior Temporal Gyrus  (-40, -16, 54)  (-58, -46, 20)  (-60, -56, 20)  Insula  (48, 8, -12)  (-44, 0, 4)  (-42, 4, -2)  (-48, -40, 24)  (40, 0, 12)  Precentral gyrus  (-56, 4, 12)  Cerebellum  (26, -54, -20)  (16, -56, -20)  (26, -62, -46)  (16, -58, -52)  Claustrum  (40, -4, 4)  Thalamus  (-12, -20, 4)  (-28, -32, 8)  (18, -10, 2)  Midbrain  (-16, -24, -4)  Parahippocampal gyrus  (-26, -32, -4)  Inferior frontal gyrus  (56, 16, 8)  (50, 24, 0)  (60, 8, 12)  Middle frontal gyrus  (6, 6, 60)  (-2, 8, 60)  (32, 0, 12)  Middle Temporal Gyrus  (21, 60, -32) |
| Copolov et al., 2003 | PET | Schizophrenia=7  Schizoaffective=1  Healthy controls=8 | Button press during AVH |  |
| Diederen et al., 2012 | fMRI | Nonpsychotic subjects with AVH=21  Non AVH psychotic patients=21  No healthy controls | Button press during AVH |  |
| Diederen et al., 2013 | fMRI | Psychotic patients with medication resistant AVH=33  No healthy controls | Button press during AVH |  |
| Dierks et al., 1999 | fMRI | Schizophrenia=3  No healthy controls | Button press during AVH |  |
| Jardri et al., 2013 | fMRI | Drug-free adolescents with a brief psychotic disorder=20  Healthy controls=20 | Button press during AVH |  |
| Lennox et al., 2000 | fMRI | Schizophrenia=4  No healthy controls | Button press during AVH |  |
| Linden et al., 2011 | fMRI | Nonclinical hallucinations=7  No healthy controls | Button press during AVH |  |
| Raij et al., 2009 | fMRI | Schizophrenia=7  Schizoaffective disorder =4  No healthy controls | Button press during AVH |  |
| Shergill et al., 2000 | fMRI | Schizophrenia=6  No healthy controls | Random sampling and button press during AVH |  |
| Shergill et al., 2004 | fMRI | Schizophrenia=2  No healthy controls | Button press during AVH |  |
| Silbersweig et al., 1995 | PET | Schizophrenia =5  Non-clinical diagnosis with auditory & visual hallucinations=1  No healthy controls | Button press during AVH |  |
| Sommer et al., 2008 | fMRI | Schizophrenia=18  Schizoaffective disorder =3  Unspecified psychotic disorder =3  No healthy controls | Button press during AVH |  |
| AVH=auditory verbal hallucinations; fMRI=functional magnetic resonance imaging; PET=Positron Emission Tomography; x=sagittal; y=coronal; z=axial; results presented as reported by Zmigrod et al (2016). | | | | |

| **Table S11. Brain regions and coordinates reported in meta-analyses of auditory verbal hallucinations (AVH)** | | | | | | |
| --- | --- | --- | --- | --- | --- | --- |
| **Study** | **Region** | **Laterality** | **BA** | **Coordinates** | | |
|  |  |  |  | **x** | **y** | **z** |
| **Coordinates associated with active AVH** | | | | | | |
| Jardri et al., 2011 | Inferior frontal gyrus | L | BA44 | -48 | 10 | 7 |
|  |  | R | BA47 | 42 | 12 | -10 |
|  | Anterior insula | L | - | -42 | 0 | 6 |
|  |  | R | - | 44 | 6 | -4 |
|  | Precentral gyrus | L | BA6 | -54 | 0 | 14 |
|  | Hippocampal complex | L | BA27 | -24 | -32 | -4 |
|  | Superior temporal gyrus | L | BA21 | -54 | -44 | 16 |
|  | Supramarginal gyrus | L | BA40 | -52 | -20 | 15 |
| Kompus et al., 2011 | Superior frontal gyrus | R | BA10 | 26 | 42 | 26 |
|  | Inferior frontal gyrus | R | BA44 | 40 | 12 | 16 |
|  | Superior temporal gyrus | L | BA42 | -52 | -22 | 16 |
|  |  | R | BA22 | 58 | -44 | 14 |
|  | Middle temporal gyrus | R | BA21 | 54 | -32 | -4 |
|  | Inferior parietal lobule | R | BA40 | 32 | -40 | 48 |
|  | Postcentral gyrus | L | BA2 | -50 | -24 | 40 |
|  | Hippocampal complex | L | - | -24 | -32 | -4 |
|  | Anterior insula | L | - | -44 | -2 | 6 |
|  | Cerebellum | R | - | 20 | -46 | -16 |
| Kühn and Gallinat, 2012 | Inferior frontal gyrus | L | BA9 | -42 | 2 | 18 |
|  | Anterior cingulate gyrus | L | BA24 | -9 | 4 | 37 |
|  |  | L | BA32 | -4 | 26 | 31 |
|  | Superior temporal gyrus | L | BA22 | -44 | -22 | 0 |
|  | Middle temporal gyrus | L | BA21 | -56 | -30 | 0 |
|  | Premotor cortex | L | BA6 | -10 | 3 | 56 |
| **Coordinates from task-related activation in patients with a history of AVH** | | | | | | |
| Kompus et al., 2011 | Superior frontal gyrus | R | BA10 | 24 | 50 | 14 |
|  | Superior temporal gyrus | L | BA22 | -54 | -8 | 0 |
|  | Anterior cingulate gyrus | L | BA24 | -10 | 0 | 40 |
|  | Hippocampal complex | L | - | -12 | -38 | 10 |
|  | Thalamus | R | - | 12 | -22 | 18 |
| Kühn and Gallinat, 2012 | Broca's area | L | BA44 | -48 | 2 | 6 |
|  | Transverse gyrus | L | BA41 | -55 | -19 | 16 |
|  | Postcentral gyrus  Postcentral gyrus | L | BA3 | -49 | -17 | 41 |
|  |  | R | BA3 | 36 | -32 | 50 |
| Zmigrod et al, 2016 | Postcentral Gyrus | L | BA3 | −46 | −18 | 44 |
|  | Postcentral Gyrus | L | BA2 | −52 | −22 | 50 |
|  | Postcentral Gyrus | L | BA3 | −40 | −16 | 54 |
|  | Superior Temporal Gyrus | R | BA22 | 54 | 12 | −6 |
|  | Insula | R | BA13 | 48 | 8 | −12 |
|  | Precentral gyrus | L | BA6 | −56 | 4 | 12 |
|  | Insula | L | BA13 | −44 | 0 | 4 |
|  | Insula | L | BA13 | −42 | 4 | −2 |
|  | Postcentral Gyrus | R | BA2 | 60 | −22 | 44 |
|  | Postcentral Gyrus | R | BA3 | 64 | −16 | 36 |
|  | Cerebellum | R | - | 26 | −54 | −20 |
|  | Cerebellum | R | - | 16 | −56 | −20 |
|  | Thalamus | L | - | −12 | −20 | 4 |
|  | Midbrain | L | - | −16 | −24 | −4 |
|  | Parahippocampal Gyrus | L | BA27 | −26 | −32 | −4 |
|  | Thalamus | L | - | −28 | −32 | 8 |
|  | Superior Temporal Gyrus | L | BA13 | −58 | −46 | 20 |
|  | Insula | L | BA13 | −48 | −40 | 24 |
|  | Superior Temporal Gyrus | L | BA22 | −60 | −56 | 20 |
|  | Inferior frontal gyrus | R | BA44 | 56 | 16 | 8 |
|  | Inferior frontal gyrus | R | BA45 | 50 | 24 | 0 |
|  | Inferior frontal gyrus | R | BA44 | 60 | 8 | 12 |
|  | Medial frontal gyrus | R | BA6 | 6 | 6 | 60 |
|  | Medial frontal gyrus | L | BA6 | −2 | 8 | 60 |
|  | Cerebellum | R | - | 26 | −62 | −46 |
|  | Cerebellum | R | - | 16 | −58 | −52 |
|  | Insula | R | BA13 | 40 | 0 | 12 |
|  | Claustrum | R | - | 40 | −4 | 4 |
|  | Medial frontal gyrus | L | BA32 | 0 | 12 | 46 |
|  | Middle Temporal Gyrus | R | BA21 | 60 | −32 | −6 |
|  | Thalamus | R | - | 18 | −10 | 2 |
| **Coordinates from brain morphological meta-analyses of patients with a history of AVH** | | | | | | |
| Modinos et al., 2012 | Superior temporal gyrus | L | BA22 | -52 | -18 | 2 |
|  |  | R | BA22 | 46 | -16 | -8 |
|  | Middle temporal gyrus | R | BA22 | 50 | -14 | -10 |
|  | Transverse gyrus | L  L | BA41 | -44 | -22 | 12 |
|  |  |  | BA22 | -46 | -14 | 6 |
|  |  | R | BA22 | 50 | -14 | 6 |
| Palaniyappan et al., 2012 | Anterior Insula | L | - | -42 | -4 | 2 |
|  | Precentral gyrus | R | BA43 | 58 | -6 | 10 |

**
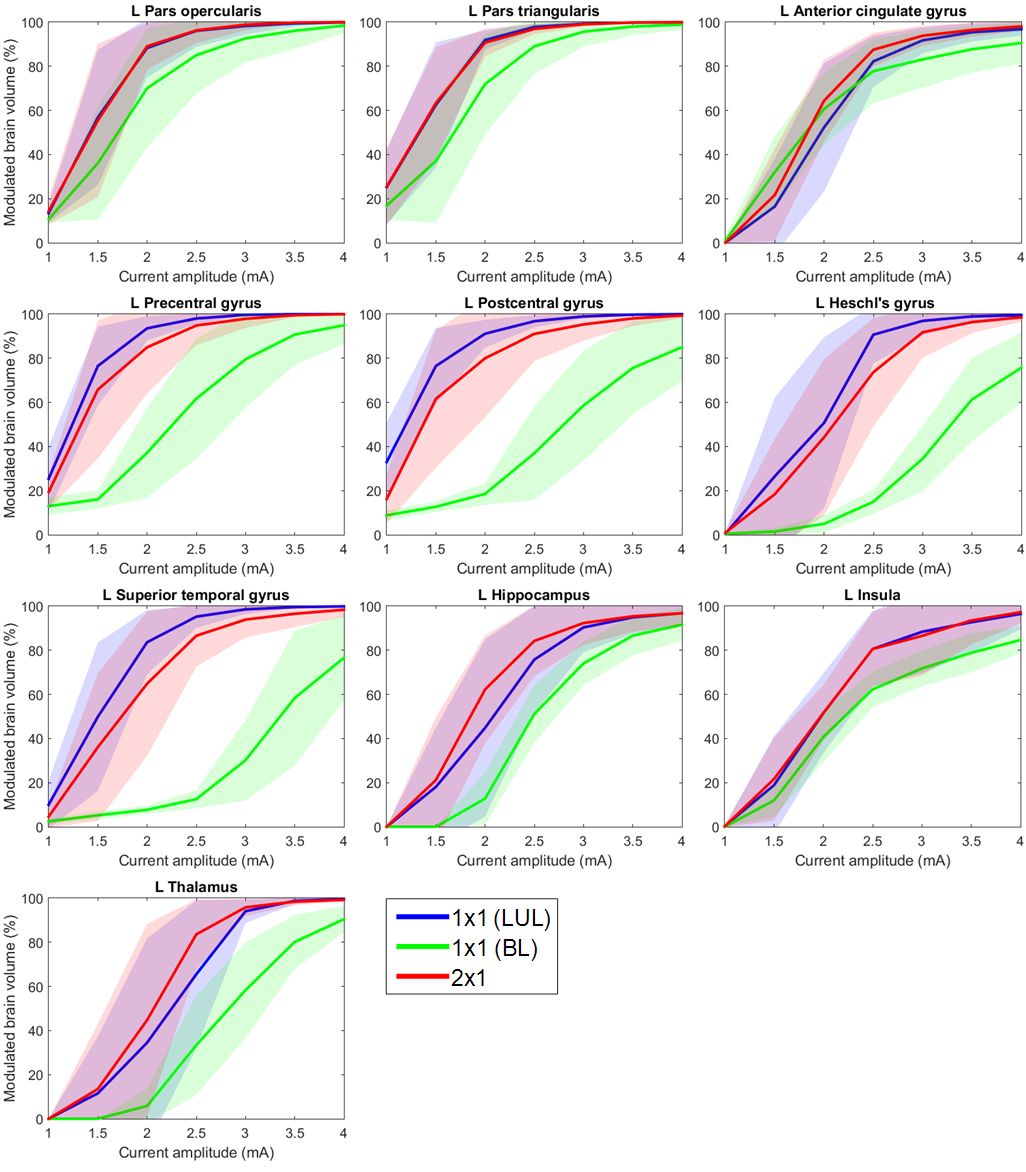
**

**Figure S1**. Percentage of modulated brain volume in each of the left AVH regions of interests (ROIs) as a function of current amplitude for the three montages. Lines and shaded regions correspond respectively to the mean and standard deviation of the modulated brain volume in each ROI across the five head models for each montage. Blue: Left unilateral (LUL) 1x1 montage; Green: Bilateral (BL) prefrontal 1x1 montage; Red: 2x1 montage; mA = milliampere; L= left.

**
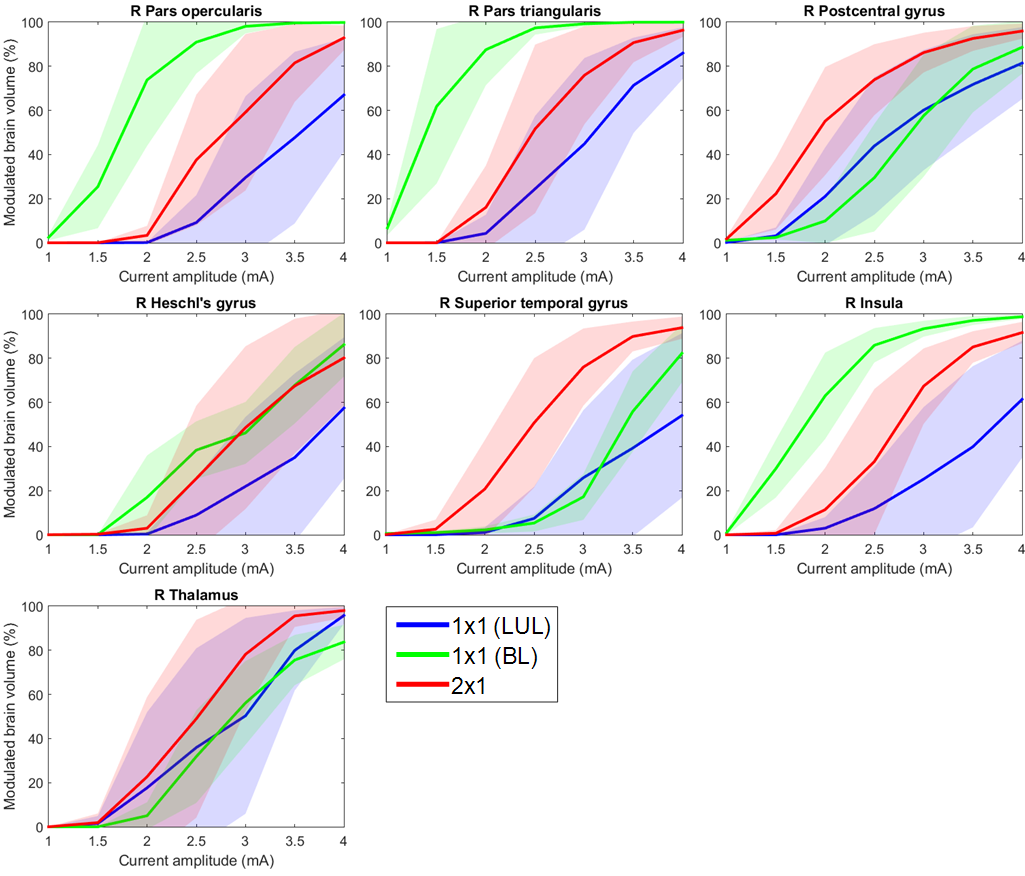
**

**Figure S2**. Percentage of modulated brain volume in each of the right-sided AVH regions of interests (ROIs) as a function of current amplitude for the three montages. Lines and shaded regions correspond respectively to the mean and standard deviation of the modulated brain volume in each ROI across the five head models for each montage. Blue: Left unilateral (LUL) 1x1 montage; Green: Bilateral (BL) prefrontal 1x1 montage; Red: 2x1 montage; mA = milliampere; R = right.

**
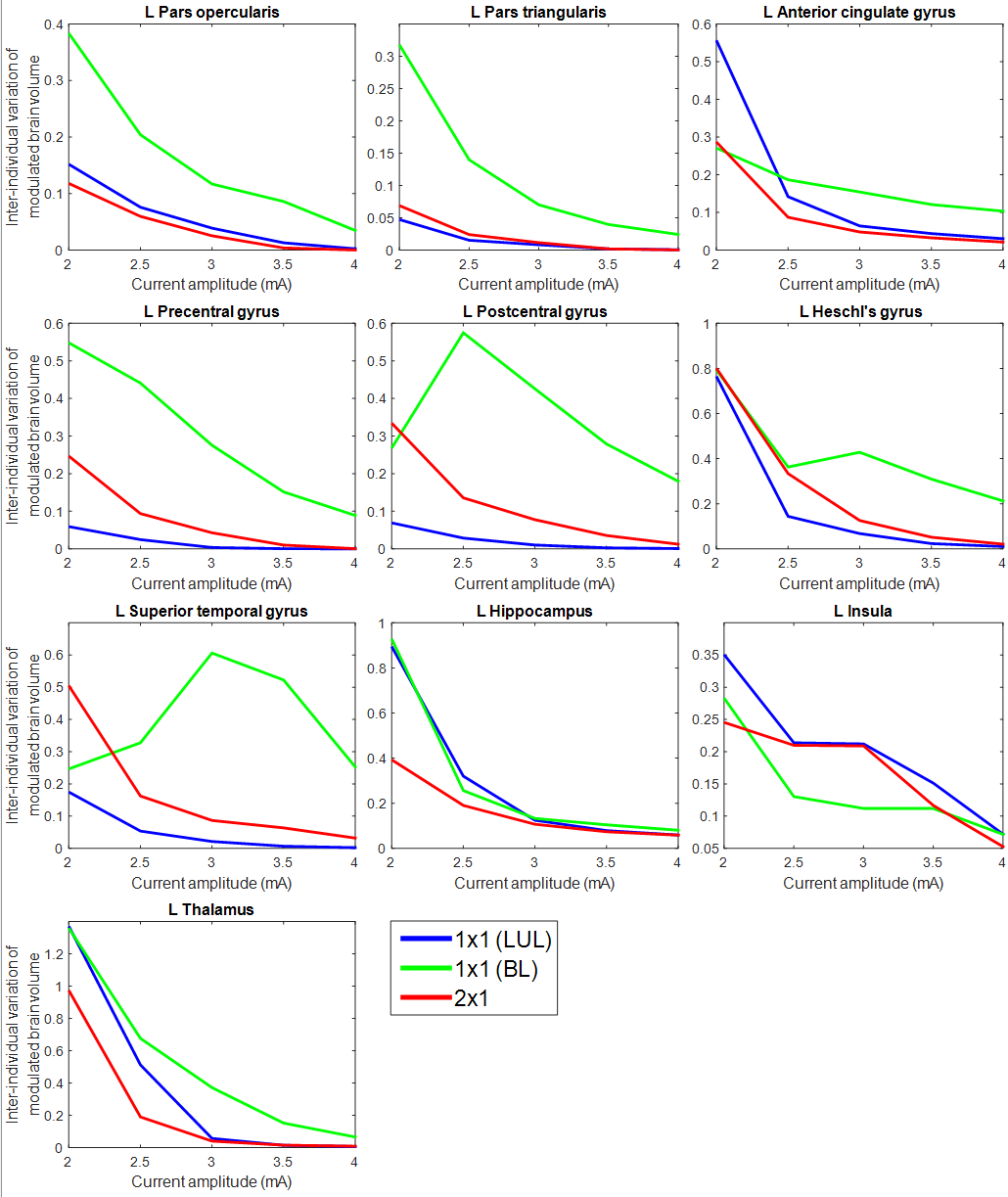
**

**Figure S3**. The coefficient of variation (CV) of the percentage modulated brain volume in each of the left-sided AVH regions of interest (ROIs) as a function of current amplitude for the three montages. Blue: Left unilateral (LUL) 1x1 montage; Green: Bilateral (BL) prefrontal 1x1 montage; Red: 2x1 montage; mA = milliampere; L = Left.

**
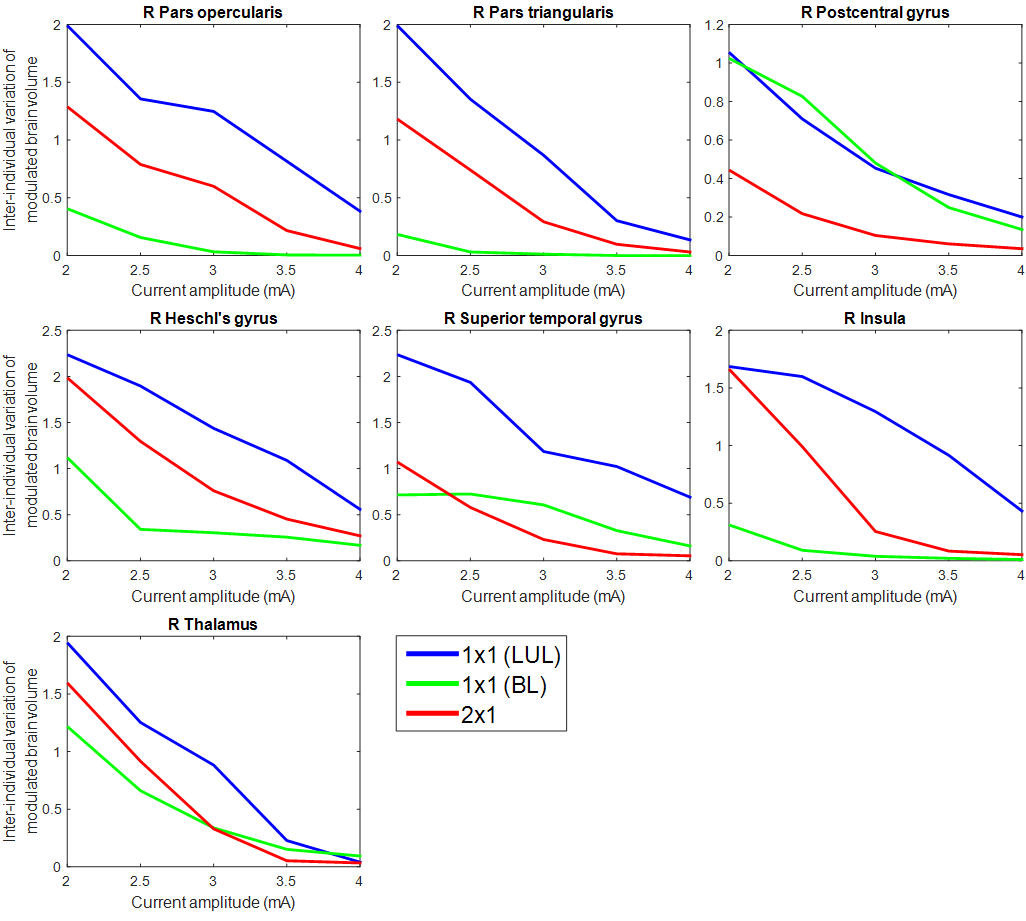
**

**Figure S4**. The coefficient of variation (CV) of the percentage modulated brain volume in each of the right-sided AVH regions of interest (ROIs) as a function of current amplitude for the three montages. Blue: Left unilateral (LUL) 1x1 montage; Green: Bilateral (BL) prefrontal 1x1 montage; Red: 2x1 montage; mA = milliampere; R = right.

**References**

Allen, P., Aleman, A., McGuire, P.K., 2007. Inner speech models of auditory verbal hallucinations: evidence from behavioural and neuroimaging studies. Int Rev Psychiatry 19, 407-415.

Andrade, C., 2013a. Transcranial direct current stimulation for refractory auditory hallucinations in schizophrenia. J. Clin. Psychiatry 74, e1054-8.

Andrade, C., 2013b. Once- to twice-daily, 3-year domiciliary maintenance transcranial direct current stimulation for severe, disabling, clozapine-refractory continuous auditory hallucinations in schizophrenia. J. ECT 29, 239–242.

Agarwal, S.M., Bose, A., Shivakumar, V., Narayanaswamy, J.C., Chhabra, H., Kalmady, S. V, Varambally, S., Nitsche, M.A., Venkatasubramanian, G., Gangadhar, B.N., 2016. Impact of antipsychotic medication on transcranial direct current stimulation (tDCS) effects in schizophrenia patients. Psychiatry Res 235, 97–103.

Barkus, E., Stirling, J., Hopkins, R., McKie, S., Lewis, S., 2007. Cognitive and neural processes in non-clinical auditory hallucinations. Br J Psychiatry Suppl 51, s76-81.

Blom, J.D., Looijestijn, J., Goekoop, R., Diederen, K.M., Rijkaart, A.M., Slotema, C.W., Sommer, I.E., 2011 Treatment of Alice in Wonderland syndrome and verbal auditory hallucinations using repetitive transcranial magnetic stimulation: a case report with fMRI findings. Psychopathology 44, 337-344.

Bose, A., Shivakumar, V., Narayanaswamy, J.C., Nawani, H., Subramaniam, A., Agarwal, S.M., Chhabra, H., Kalmady, S. V, Venkatasubramanian, G., 2014. Insight facilitation with add-on tDCS in schizophrenia. Schizophr Res 156, 63–65.

Bose, A., Sowmya, S., Shenoy, S., Agarwal, S.M., Chhabra, H., Narayanaswamy, J.C., Venkatasubramanian, G., 2015. Clinical utility of attentional salience in treatment of auditory verbal hallucinations in schizophrenia using transcranial direct current stimulation (tDCS). Schizophr Res 164, 279–80.

Braus, D.F., Weber-Fahr, W., Tost, H., Ruf, M., Henn, F.A., 2002. Sensory information processing in neuroleptic-naive first-episode schizophrenic patients: a functional magnetic resonance imaging study. Arch Gen Psychiatry 59, 696-701.

Brunelin, J., Mondino, M., Gassab, L., Haesebaert, F., Gaha, L., Suaud-Chagny, M.-F., Saoud, M., Mechri, A., Poulet, E., 2012. Examining Transcranial Direct-Current Stimulation (tDCS) as a Treatment for Hallucinations in Schizophrenia. Am J Psychiatry 169, 719–724.

Copolov, D.L., Seal, M.L., Maruff, P., Ulusoy, R., Wong, M.T., Tochon-Danguy, H.J., Egan, G.F., 2003. Cortical activation associated with the experience of auditory hallucinations and perception of human speech in schizophrenia: a PET correlation study. Psych Research 122,139-152.

Diederen, K.M., Neggers, S.F., Daalman, K., Blom, J.D., Goekoop, R., Kahn, R.S., Sommer, I.E., 2010. Deactivation of the parahippocampal gyrus preceding auditory hallucinations in schizophrenia. Am J Psychiatry 167,427-435.

Diederen, K.M., Daalman, K,, de Weijer, A.D., Neggers, S.F., van Gastel, W., Blom, J.D., Kahn, R.S., Sommer, I.E., 2012. Auditory hallucinations elicit similar brain activation in psychotic and nonpsychotic individuals. Schizophr Bull 38, 1074-1082.

Diederen, K.M., Charbonnier, L., Neggers, S.F., van Lutterveld, R., Daalman, K., Slotema, C.W., Kahn, R.S., Sommer, I.E., 2013. Reproducibility of brain activation during auditory verbal hallucinations. Schizophr Res 146, 320-325.

Dierks, T., Linden, D.E., Jandl, M., Formisano, E., Goebel, R., Lanfermann, H., Singer, W., 1999. Activation of Heschl's gyrus during auditory hallucinations. Neuron 22, 615-621.

Fitzgerald, P.B., McQueen, S., Daskalakis, Z.J., Hoy, K.E., 2014. A Negative Pilot Study of Daily Bimodal Transcranial Direct Current Stimulation in Schizophrenia. Brain Stimul 7, 813–816.

Fröhlich, F., Burrello, T.N., Mellin, J.M., Cordle, A.L., Lustenberger, C.M., Gilmore, J.H., Jarskog, L.F., 2016. Exploratory study of once-daily transcranial direct current stimulation (tDCS) as a treatment for auditory hallucinations in schizophrenia. Eur Psychiatry 33, 54–60.

Ganguli, R., Carter, C., Mintun, M., Brar, J., Becker, J., Sarma, R., Nichols, T., Bennington, E., 1997. PET brain mapping study of auditory verbal supraspan memory versus visual fixation in schizophrenia. Biol Psychiatry 41, 33-42.

García-Martí, G., Aguilar, E.J., Lull, J.J., Martí-Bonmatí, L., Escartí, M.J., Manjón, J.V., Moratal, D., Robles, M., Sanjuán J., 2008. Schizophrenia with auditory hallucinations: a voxel-based morphometry study. Prog Neuropsychopharmacol Biol Psychiatry 32, 72-80.

Gaser, C., Nenadic, I., Volz, H.P., Büchel, C., Sauer, H., 2004. Neuroanatomy of "hearing voices": a frontotemporal brain structural abnormality associated with auditory hallucinations in schizophrenia. Cereb Cortex 14, 91-96.

Haddock, G., McCarron, J., Tarrier, N., Faragher, E.B., 1999. Scales to measure dimensions of hallucinations and delusions: the psychotic symptom rating scales (PSYRATS). Psychol Med 29, 879–89.

Hoffman, R.E., Anderson, A.W., Varanko, M., Gore, J.C., Hampson, M., 2008. Time course of regional brain activation associated with onset of auditory/verbal hallucinations. Br J Psychiatry 193, 424-425.

Homan, P., Kindler, J., Federspiel, A., Flury, R., Hubl, D., Hauf, M., Dierks, T., 2011. Muting the voice: A case of arterial spin labeling-monitored transcranial direct current stimulation treatment of auditory verbal hallucinations. Am J Psychiatry 168, 853–854.

Jardri, R., Pins, D., Delmaire, C., Goeb, J.L., Thomas, P., 2007. Activation of bilateral auditory cortex during verbal hallucinations in a child with schizophrenia. Mol Psychiatry 12, 319.

Jardri R., Pins, D., Bubrovszky, M., Lucas, B., Lethuc, V., Delmaire, C., Vantyghem, V., Despretz, P., Thomas, P., 2009. Neural functional organization of hallucinations in schizophrenia: multisensory dissolution of pathological emergence in consciousness. Conscious Cogn 18, 449-457.

Jardri, R., Pouchet, A., Pins, D., Thomas, P., 2011. Cortical activations during auditory verbal hallucinations in schizophrenia: a coordinate-based meta-analysis. Am J Psychiatry 168, 73–81.

Jardri, R., Thomas, P., Delmaire, C., Delion, P., Pins, D., 2013. The neurodynamic organization of modality-dependent hallucinations. Cereb Cortex 23,1108-1117.

Kang, J.I., Kim, J.J., Seok, J.H., Chun, J.W., Lee, S.K., Park, H.J., 2009. Abnormal brain response during the auditory emotional processing in schizophrenic patients with chronic auditory hallucinations. Schizophr Res 107, 83-91.

Kay SR, Fiszbein A, Opler LA. The positive and negative syndrome scale (PANSS) for schizophrenia. Schizophr Bull 1987;13:261-76

Koeda, M., Takahashi, H., Yahata, N., Matsuura, M., Asai, K., Okubo, Y., Tanaka, H., 2006. Language processing and human voice perception in schizophrenia: a functional magnetic resonance imaging study. Biol Psychiatry 59, 948-957.

Kompus, K., Westerhausen, R., Hugdahl, K., 2011. The “paradoxical” engagement of the primary auditory cortex in patients with auditory verbal hallucinations: A meta-analysis of functional neuroimaging studies. Neuropsychologia 49, 3361–3369.

Kühn, S., Gallinat, J., 2012. Quantitative meta-analysis on state and trait aspects of auditory verbal hallucinations in schizophrenia. Schizophr Bull 38, 779–786.

Kumari, V., Fannon, D., Ffytche, D.H., Raveendran, V., Antonova, E., Premkumar, P., Cooke, M.A., Anilkumar, A.P., Williams, S.C., Andrew, C., Johns, L.C., Fu, C.H., McGuire, P.K., Kuipers, E., 2010. Functional MRI of verbal self-monitoring in schizophrenia: performance and illness-specific effects. Schizophr Bull 36, 740-755.

Lennox, B.R., Park, S.B., Jones, P.B., Morris, P.G., 1999. Spatial and temporal mapping of neural activity associated with auditory hallucinations. Lancet 353:644.

Lennox, B.R., Park, S.B., Medley, I., Morris, P.G. Jones, P.B., 2000. The functional anatomy of auditory hallucinations in schizophrenia. Psychiatry Res 100, 13-20.

Linden, D.E., Thornton, K., Kuswanto, C.N., Johnston, S.J., van de Ven, V., Jackson, M.C., 2011. The brain's voices: comparing nonclinical auditory hallucinations and imagery. Cereb Cortex 21, 330-337.

Mattai, A., Miller, R., Weisinger, B., Greenstein, D., Bakalar, J., Tossell, J., David, C., Wassermann, E.M., Rapoport, J., Gogtay, N., 2011. Tolerability of transcranial direct current stimulation in childhood-onset schizophrenia. Brain Stimul 4, 275–280.

McGuire, P.K., Silbersweig, D.A., Wright, I., Murray, R.M., David, A.S., Frackowiak, R.S., Frith, C.D., 1995. Abnormal monitoring of inner speech: a physiological basis for auditory hallucinations. Lancet 346, 596-600.

Mitchell, R.L., Elliott, R., Barry, M., Cruttenden, A., Woodruff, P.W., 2004. Neural response to emotional prosody in schizophrenia and in bipolar affective disorder. Br J Psychiatry 184, 223-230.

Mondino, M., Brunelin, J., Palm, U., Brunoni, A.R., Poulet, E., Fecteau, S., 2015. Transcranial direct current stimulation for the treatment of refractory symptoms of schizophrenia. Current evidence and future directions. Curr Pharm Des 21, 3373–3383.

Mondino, M., Haesebaert, F., Poulet, E., Suaud-Chagny, M.F., Brunelin, J., 2014. Fronto-temporal transcranial Direct Current Stimulation (tDCS) reduces source-monitoring deficits and auditory hallucinations in patients with schizophrenia. Schizophr Res 161, 54–55.

Modinos, G., Vercammen, A., Mechelli, A., Knegtering, H., McGuire, P.K., Aleman, A., 2009. Structural covariance in the hallucinating brain: a voxel-based morphometry study. J Psychiatry Neurosci 34, 465-469.

Modinos, G., Costafreda, S.G., Van Tol, M.J., McGuire, P.K., Aleman, A., Allen, P., 2013. Neuroanatomy of auditory verbal hallucinations in schizophrenia: A quantitative meta-analysis of voxel-based morphometry studies. Cortex 49, 1046–1055.

Morey, R.A., Mitchell, T.V., Inan, S., Lieberman, J.A., Belger, A., 2008. Neural correlates of automatic and controlled auditory processing in schizophrenia. J Neuropsychiatry Clin Neurosci 20, 419-430.

Narayanaswamy, J.C., Shivakumar, V., Bose, A., Agarwal, S.M., Venkatasubramanian, G., Gangadhar, B.N., 2014. Sustained improvement of negative symptoms in schizophrenia with add-on tDCS: a case report. Clin Schizophr Relat Psychoses 8, 135–6.

Nawani, H., Bose, A., Agarwal, S.M., Shivakumar, V., Chhabra, H., Subramaniam, A., Kalmady, S., Narayanaswamy, J.C., Venkatasubramanian, G., 2014a. Modulation of corollary discharge dysfunction in schizophrenia by tDCS: preliminary evidence. Brain Stimul 7, 486–488.

Nawani, H., Kalmady, S. V, Bose, A., Shivakumar, V., Rakesh, G., Subramaniam, A., Narayanaswamy, J.C., Venkatasubramanian, G., 2014b. Neural basis of tDCS effects on auditory verbal hallucinations in schizophrenia: a case report evidence for cortical neuroplasticity modulation. J ECT 30, e2-4.

Neckelmann, G., Specht, K., Lund, A., Ersland, L., Smievoll, A.I., Neckelmann, D., Hugdahl, K., 2006. MR morphometry analysis of grey matter volume reduction in schizophrenia: association with hallucinations. Int J Neurosci 116, 9-23.

Nenadic, I., Smesny, S., Schlösser, R.G., Sauer, H, Gaser, C., 2010. Auditory hallucinations and brain structure in schizophrenia: voxel-based morphometric study. Br J Psychiatry 196, 412-413.

Ngan, E.T., Vouloumanos, A., Cairo, T.A., Laurens, K.R., Bates, A.T., Anderson, C.M., Werker, J.F., Liddle, P.F., 2003. Abnormal processing of speech during oddball target detection in schizophrenia. Neuroimage 20, 889-897.

O'Daly, O.G., Frangou, S., Chitnis, X., Shergill, S.S., 2007. Brain structural changes in schizophrenia patients with persistent hallucinations. Psychiatry Res 156, 15-21.

Ojeda, N., Ortuño, F., Arbizu, J., López, P., Martí-Climent, J.M., Peñuelas, I., Cervera-Enguix, S., 2002. Functional neuroanatomy of sustained attention in schizophrenia: contribution of parietal cortices. Hum Brain Mapp 17, 116-130.

Palaniyappan, L., Balain, V., Radua, J., Liddle, P.F., 2012. Structural correlates of auditory hallucinations in schizophrenia: A meta-analysis. Schizophr Res 137, 169–173.

Plaze, M., Bartrés-Faz, D., Martinot, J.L., Januel, D., Bellivier, F., De Beaurepaire, R., Chanraud, S., Andoh, J., Lefaucheur, J.P., Artiges, E., Pallier, C., Paillère-Martinot, M.L., 2006. Left superior temporal gyrus activation during sentence perception negatively correlates with auditory hallucination severity in schizophrenia patients. Schizophr Res 87, 109-115.

Praharaj, S.K., Behere, R. V, Sharma, P.S.V.N., 2015. Cathodal Transcranial Direct Current Stimulation Over Left Temporoparietal Area for Treatment-Refractory Delusions and Auditory Hallucinations in Schizophrenia: A Case Study. J ECT 31, 277–278.

Raij, T.T., Valkonen-Korhonen, M., Holi, M., Therman, S., Lehtonen, J., Hari, R., 2009. Reality of auditory verbal hallucinations. Brain 132:2994-3001.

Rakesh, G., Shivakumar, V., Subramaniam, A., Nawani, H., Amaresha, A.C., Narayanaswamy, J.C., Venkatasubramanian, G., 2013. Monotherapy with tDCS for schizophrenia: A case report. Brain Stimul 6, 708–709.

Shapleske, J., Rossell, S.L., Chitnis, X.A., Suckling, J., Simmons, A., Bullmore, E.T., Woodruff, P.W., David, A.S., 2002. A computational morphometric MRI study of schizophrenia: effects of hallucinations. Cereb Cortex 12, 1331-1341.

Shenoy, S., Bose, A., Chhabra, H., Dinakaran, D., Agarwal, S.M., Shivakumar, V., Narayanaswamy, J.C., Sivakumar, P.T., Venkatasubramanian, G., 2015. Transcranial Direct Current Stimulation (tDCS) for Auditory Verbal Hallucinations in Schizophrenia During Pregnancy: A Case Report. Brain Stimul 8, 163–164.

Shergill, S.S., Brammer, M.J., Williams, S.C., Murray, R.M., McGuire, P.K., 2000. Mapping auditory hallucinations in schizophrenia using functional magnetic resonance imaging. Arch Gen Psychiatry 57, 1033-1038.

Shergill, S.S., Bullmore, E.T., Brammer, M.J., Williams, S.C., Murray, R.M., McGuire, P.K., 2001. A functional study of auditory verbal imagery. Psychol Med 31, 241-253.

Shergill, S.S., Brammer, M.J., Amaro, E., Williams, S.C., Murray, R.M., McGuire, P.K., 2004. Temporal course of auditory hallucinations. Br J Psychiatry185, 516-517.

Shiozawa, P., da Silva, M.E., Cordeiro, Q., Fregni, F., Brunoni, A.R., 2013. Transcranial direct current stimulation (tDCS) for the treatment of persistent visual and auditory hallucinations in schizophrenia: a case study. Brain Stimul 6, 831–833.

Shivakumar, V., Bose, A., Rakesh, G., Nawani, H., Subramaniam, A., Agarwal, S.M., Kalmady, S. V, Narayanaswamy, J.C., Venkatasubramanian, G., 2013. Rapid improvement of auditory verbal hallucinations in schizophrenia after add-on treatment with transcranial direct-current stimulation. J ECT 29, e43-4.

Shivakumar, V., Chhabra, H., Subbanna, M., Agarwal, S.M., Bose, A., Kalmady, S. V, Narayanaswamy, J.C., Debnath, M., Venkatasubramanian, G., 2015. Effect of tDCS on auditory hallucinations in schizophrenia: Influence of catechol-O-methyltransferase (COMT) Val158Met polymorphism. Asian J Psychiatr 16, 75–7.

Shivakumar, V., Narayanaswamy, J.C., Agarwal, S.M., Bose, A., Subramaniam, A., Venkatasubramanian, G., 2014. Targeted, intermittent booster tDCS: A novel add-on application for maintenance treatment in a schizophrenia patient with refractory auditory verbal hallucinations. Asian J Psychiatr 11, 79–80.

Silbersweig, D.A., Stern, E., Frith, C., Cahill, C., Holmes, A., Grootoonk, S., Seaward, J., McKenna, P., Chua, S.E., Schnorr L., Jones, T., Frackowiak, R.S.J., 1995. A functional neuroanatomy of hallucinations in schizophrenia. Nature 378,176-179.

Simons, C.J., Tracy, D.K., Sanghera, K.K., O'Daly, O., Gilleen, J., Dominguez, M.D., Krabbendam, L., Shergill, S.S., 2010. Functional magnetic resonance imaging of inner speech in schizophrenia. Biol Psychiatry 67, 232-237.

Smith, R.C., Boules, S., Mattiuz, S., Youssef, M., Tobe, R.H., Sershen, H., Lajtha, A., Nolan, K., Amiaz, R., Davis, J.M., 2015. Effects of transcranial direct current stimulation (tDCS) on cognition, symptoms, and smoking in schizophrenia: A randomized controlled study. Schizophr Res 168, 260–266.

Sommer, I.E., Diederen, K.M., Blom, J.D., Willems, A., Kushan, L., Slotema, K., Boks, M.P., Daalman, K., Hoek, H.W., Neggers, S.F., Kahn, R.S., 2008. Auditory verbal hallucinations predominantly activate the right inferior frontal area. Brain 131, 3169-3177.

Stephane, M., Hagen, M.C., Lee, J.T., Uecker, J., Pardo, P.J., Kuskowski, M.A., Pardo, J.V., 2006. About the mechanisms of auditory verbal hallucinations: a positron emission tomographic study. J Psychiatry Neurosci 31, 396-3405.

Subramaniam, A., Agarwal, S.M., Kalmady, S., Shivakumar, V., Chhabra, H., Bose, A., Damodharan, D., Narayanaswamy, J.C., Hutton, S.B., Venkatasubramanian, G., 2015. Effect of Transcranial Direct Current Stimulation on Prefrontal Inhibition in Schizophrenia Patients with Persistent Auditory Hallucinations: A Study on Antisaccade Task Performance. Indian J Psychol. Med 37, 419-422.

Woodruff, P.W., Wright, I.C., Bullmore, E.T., Brammer, M., Howard, R.J., Williams, S.C., Shapleske, J., Rossell, S., David, A.S., McGuire, P.K., Murray, R.M., 1997. Auditory hallucinations and the temporal cortical response to speech in schizophrenia: a functional magnetic resonance imaging study. Am J Psychiatry 154, 1676-1682.

van Tol, M.J., van der Meer, L., Bruggeman, R., Modinos, G., Knegtering, H., Aleman, A., 2013. Voxel-based gray and white matter morphometry correlates of hallucinations in schizophrenia: The superior temporal gyrus does not stand alone. Neuroimage Clin. 4, 249-257.

Zhang, Z., Shi, J., Yuan, Y., Hao, G., Yao, Z., Chen, N., 2008. Relationship of auditory verbal hallucinations with cerebral asymmetry in patients with schizophrenia: an event-related fMRI study. J Psychiatr Res 42, 477-486.

Zmigrod, L., Garrison, J.R., Carr, J., Simons, J.S., 2016. The neural mechanisms of hallucinations: A quantitative meta-analysis of neuroimaging studies. Neurosci Biobehav Rev 69,113-123.
